# Antimalarial chemoprophylaxis for forest goers in southeast Asia: an open-label, individually randomised controlled trial

**DOI:** 10.1016/S1473-3099(22)00492-3

**Published:** 2023-01

**Authors:** Rupam Tripura, Lorenz von Seidlein, Siv Sovannaroth, Thomas J Peto, James J Callery, Meas Sokha, Mom Ean, Chhouen Heng, Franca Conradis-Jansen, Wanassanan Madmanee, Pimnara Peerawaranun, Naomi Waithira, Panarasri Khonputsa, Monnaphat Jongdeepaisal, Kulchada Pongsoipetch, Paphapisa Chotthanawathit, Ung Soviet, Christopher Pell, Jureeporn Duanguppama, Huy Rekol, Joel Tarning, Mallika Imwong, Mavuto Mukaka, Nicholas J White, Arjen M Dondorp, Richard J Maude

**Affiliations:** a Mahidol Oxford Tropical Medicine Research Unit, Faculty of Tropical Medicine, Mahidol University, Bangkok, Thailand; b Department of Molecular Tropical Medicine and Genetics, Faculty of Tropical Medicine, Mahidol University, Bangkok, Thailand; c Centre for Tropical Medicine and Global Health, Nuffield Department of Clinical Medicine, University of Oxford, Oxford, UK; d National Center for Parasitology, Entomology and Malaria Control, Phnom Penh, Cambodia; e University of Heidelberg, Heidelberg, Germany; f Stung Treng Provincial Health Department, Stung Treng, Cambodia; g Amsterdam Institute for Global Health and Development (AIGHD), Amsterdam, Netherlands; h Department of Global Health, Amsterdam University Medical Centers, location Academic Medical Center, and Centre for Social Science and Global Health, University of Amsterdam, Amsterdam, Netherlands; i Open University, Milton Keynes, UK; j Harvard T H Chan School of Public Health, Harvard University, Boston, MA, USA

## Abstract

**Background:**

Malaria in the eastern Greater Mekong subregion has declined to historic lows. Countries in the Greater Mekong subregion are accelerating malaria elimination in the context of increasing antimalarial drug resistance. Infections are now increasingly concentrated in remote, forested foci. No intervention has yet shown satisfactory efficacy against forest-acquired malaria. The aim of this study was to assess the efficacy of malaria chemoprophylaxis among forest goers in Cambodia.

**Methods:**

We conducted an open-label, individually randomised controlled trial in Cambodia, which recruited participants aged 16–65 years staying overnight in forests. Participants were randomly allocated 1:1 to antimalarial chemoprophylaxis, a 3-day course of twice-daily artemether–lumefantrine followed by the same daily dosing once a week while travelling in the forest and for a further 4 weeks after leaving the forest (four tablets per dose; 20 mg of artemether and 120 mg of lumefantrine per tablet), or a multivitamin with no antimalarial activity. Allocations were done according to a computer-generated randomisation schedule, and randomisation was in permuted blocks of size ten and stratified by village. Investigators and participants were not masked to drug allocation, but laboratory investigations were done without knowledge of allocation. The primary outcome was a composite endpoint of either clinical malaria with any *Plasmodium* species within 1–28, 29–56, or 57–84 days, or subclinical infection detected by PCR on days 28, 56, or 84 using complete-case analysis of the intention-to-treat population. Adherence to study drug was assessed primarily by self-reporting during follow-up visits. Adverse events were assessed in the intention-to-treat population as a secondary endpoint from self-reporting at any time, plus a physical examination and symptom questionnaire at follow-up. This trial is registered at ClinicalTrials.gov (NCT04041973) and is complete.

**Findings:**

Between March 11 and Nov 20, 2020, 1480 individuals were enrolled, of whom 738 were randomly assigned to artemether–lumefantrine and 742 to the multivitamin. 713 participants in the artemether–lumefantrine group and 714 in the multivitamin group had a PCR result or confirmed clinical malaria by rapid diagnostic test during follow-up. During follow-up, 19 (3%, 95% CI 2–4) of 713 participants had parasitaemia or clinical malaria in the artemether–lumefantrine group and 123 (17%, 15–20) of 714 in the multivitamin group (absolute risk difference 15%, 95% CI 12–18; p<0·0001). During follow-up, there were 166 malaria episodes caused by *Plasmodium vivax*, 14 by *Plasmodium falciparum*, and five with other or mixed species infections. The numbers of participants with *P vivax* were 18 (3%, 95% CI 2–4) in the artemether–lumefantrine group versus 112 (16%, 13–19) in the multivitamin group (absolute risk difference 13%, 95% CI 10–16; p<0·0001). The numbers of participants with *P falciparum* were two (0·3%, 95% CI 0·03–1·01) in the artemether–lumefantrine group versus 12 (1·7%, 0·9–2·9) in the multivitamin group (absolute risk difference 1·4%, 95% CI 0·4–2·4; p=0·013). Overall reported adherence to the full course of medication was 97% (95% CI 96–98; 1797 completed courses out of 1854 courses started) in the artemether–lumefantrine group and 98% (97–98; 1842 completed courses in 1885 courses started) in the multivitamin group. Overall prevalence of adverse events was 1·9% (355 events in 18 806 doses) in the artemether–lumefantrine group and 1·1% (207 events in 19 132 doses) in the multivitamin group (p<0·0001).

**Interpretation:**

Antimalarial chemoprophylaxis with artemether–lumefantrine was acceptable and well tolerated and substantially reduced the risk of malaria. Malaria chemoprophylaxis among high-risk groups such as forest workers could be a valuable tool for accelerating elimination in the Greater Mekong subregion.

**Funding:**

The Global Fund to Fight AIDS, Tuberculosis and Malaria; Wellcome Trust.

**Research in context**

**Evidence before this study**

We searched PubMed for articles in English published from database inception until June 30, 2022, using the terms antimalarial AND prophylaxis AND malaria, filtering the search for Clinical Trials, Meta-Analyses, Randomized Controlled Trial, and Humans, and screening for clinical trials and systematic reviews. The search resulted in 104 articles. Of these, 19 described systematic reviews and meta-analyses. Of the remaining 85 describing clinical trials, five were controlled human challenge models; 52 presented intermittent preventive treatment in infants, children, or pregnant women; four assessed seasonal malaria chemoprophylaxis; and 17 investigated the malaria-prophylactic effects of co-trimoxazole or antiretrovirals in people with HIV (n=13), travellers (n=2), or people with sickle cell anaemia (n=2). Of the remaining seven studies, one in Kenyan school children found proguanil to be effective in preventing falciparum malaria. Five trials assessed chemoprophylaxis efficacy in Asian adults, including two placebo-controlled trials of monthly, single-dose naphthoquine–azithromycin in a low-risk setting on the China–Myanmar border that showed more than 90% efficacy in preventing malaria, with a good safety profile and tolerability and follow-up periods of 1 month and 2 months. In Thailand, a trial in men with occupational risk of malaria who received monthly or bimonthly 3-day treatment courses of dihydroartemisinin–piperaquine or placebo found 98% protective efficacy and good tolerability but required close supervision with weekly follow-up. This trial was done during a period of relatively high parasite prevalence. A trial of chloroquine chemoprophylaxis in pregnancy in Thailand found it to be completely effective in preventing *Plasmodium vivax* episodes. A comparison of tafenoquine and mefloquine prophylaxis in Australian soldiers returning from Timor-Leste demonstrated safety and tolerability but could not quantify impact in preventing malaria. One trial among Thai-Burmese children found proguanil to have poor prophylactic efficacy. These trials used microscopy to detect parasites, a method with poor sensitivity for low-density infections.

**Added value of this study**

This study reports the efficacy of antimalarial chemotherapy in a low-transmission setting deliberately designed to be feasible for scale-up for routine use as an elimination intervention. Unlike previous chemoprophylaxis trials, it used low-intensity, monthly follow-up and peer supervision of dosing between follow-up visits as many of the target population spent long periods away from the village. It is the first trial to target forest goers, only recruiting those at highest risk of malaria to maximise efficiency of the intervention. Choice of antimalarials was limited to the relatively short-acting regimen of weekly artemether–lumefantrine; previous trials have used drugs with less frequent dosing and longer half-lives, which might affect adherence. PCR was used to detect parasites, as it has higher sensitivity than microscopy. Despite the low-intensity monitoring, the chemoprophylaxis had high efficacy in preventing malaria, and adherence to the study drug was high. This study provides much-needed evidence for the efficacy, tolerability, and pragmatic implementation of antimalarial chemoprophylaxis in high-risk mobile populations in real-world settings.

**Implications of all the available evidence**

This study adds to existing evidence for the efficacy and tolerability of antimalarial chemoprophylaxis in groups at high risk of malaria in low-endemic settings. It demonstrates that chemoprophylaxis is effective to prevent malaria in forest goers and can be safely managed with relatively low-intensity monthly follow-up through close engagement with, and involvement of, the local community. Malaria chemoprophylaxis among high-risk groups such as forest goers could be a valuable additional tool for malaria elimination in the Greater Mekong subregion.

## Introduction

Increasing artemisinin-resistant and multidrug-resistant malaria has been a major threat for the five countries of the Greater Mekong subregion (Viet Nam, Cambodia, Laos, Thailand, and Myanmar). This situation has led to these countries committing to eliminate *Plasmodium falciparum* malaria by 2023 and all malaria by 2030.^1^ Comprehensive national plans for malaria elimination have been implemented in all countries in the Greater Mekong subregion, resulting in a reduction in malaria incidence of more than 70% from 2015 to 2019,^2^ and almost 40% from 2019 to 2021.^3^ In Cambodia, malaria incidence has reached very low levels, with fewer than 5000 cases reported in 2021, of which around 90% were caused by *Plasmodium vivax* and 10% by *P falciparum*.^4^ In much of the Greater Mekong subregion, including Cambodia, the remaining malaria transmission is concentrated in forested areas.^5^, ^6^ Addressing the remaining parasite reservoir in forest goers is essential to reach the goal of malaria elimination within the proposed time frame.^7^ A range of interventions targeting forest goers has been proposed, including mobile volunteerS providing malaria diagnostics and treatment, standby treatment or chemoprophylaxis, long-lasting insecticide-treated hammock nets, topical repellents, and insecticide-treated clothing.^7^

Because a large proportion of the *Plasmodium* reservoir in forest goers is in asymptomatic carriers with low parasitaemias, an approach of mass screening and treatment or reactive case detection using conventional rapid diagnostic tests (RDTs) captures only a minority of infections.^6^, ^8^ The main *Anopheles* malaria vectors in the region, *Anopheles dirus, Anopheles minimus*, and *Anopheles maculatus,* tend to bite outside and before bedtime.^9^ In this epidemiological context, insecticidal nets have limited efficacy.^10^ Long-lasting insecticidal hammock nets showed only a modest protective effect in forest villages in Cambodia.^9^ The quality of the housing available in forests is limited by the available resources consisting often of campsites covered by a suspended tarpaulin canopy that is poorly suited to hanging bed nets.^11^ Several studies have shown poor use of personal protection measures against malaria transmission, including in the forest.^12^, ^13^ In the absence of simple, effective, and affordable vector-control interventions, providing forest goers with effective antimalarial chemoprophylaxis is a promising approach to protect them against malaria.^5^

We report here the results of an open-label, individually randomised controlled trial to assess the efficacy of malaria chemoprophylaxis among forest goers in Cambodia. Artemether–lumefantrine was chosen as the intervention drug because of its excellent safety profile established over more than 20 years of extensive use. In addition, although it remains an efficacious antimalarial, artemether–lumefantrine has not been used as a first-line drug in Cambodia. A disadvantage of artemether–lumefantrine is the short duration of its post-treatment prophylactic effect, related to the elimination of lumefantrine (terminal elimination half-life 4–6 days).^14^ Furthermore, artemether–lumefantrine needs to be taken with a fat-containing drink or snack, which might not always be available, to optimise the absorption of the lumefantrine. Alternative antimalarials have other disadvantages. In Cambodia, widespread resistance in *P falciparum* to both artemisinins and piperaquine precludes use of dihydroartemisinin–piperaquine.^15^ Artesunate–pyronaridine is a recently developed artemisinin-based combination therapy (ACT) approved for the treatment of falciparum malaria in the Greater Mekong subregion and the first-line treatment in some areas. However, its safety profile for extensive, unsupervised use as a prophylactic treatment has not been established.^16^ Artesunate–mefloquine is currently the first-line treatment for both falciparum and vivax malaria in Cambodia but ideally should not be used as a prophylactic to avoid additional drug pressure on the parasite population facilitating mefloquine resistance. Although tolerability of weekly prophylactic dosing is reasonable, adverse effects preclude giving treatment doses of mefloquine to healthy individuals.^17^

## Methods

### Study design and participants

We conducted an open-label, individually randomised controlled trial to compare the efficacy of malaria chemoprophylaxis with artemether–lumefantrine versus multivitamin among forest goers at 15 villages in Siem Pang District, Stung Treng province in northeastern Cambodia along the border with southern Laos. The selected district had the highest malaria incidences in Cambodia during 2018–19 at 142·3 cases per 1000 population in 2018 and 86·5 in 2019, and has high forest cover.

Enrolment was between March 11 and Nov 20, 2020, with follow-up until Feb 17, 2021. Participants aged 16–65 years who planned to travel to the forest within the next 72 h and stay overnight, and who were willing and able to comply with the study protocol, were included in the study. Excluded from enrolment were women with known pregnancy or breastfeeding or who planned to become pregnant; individuals who had received ACT within the previous 7 days; individuals with a history of allergy or known contraindication to artemisinins, lumefantrine, or multivitamins; individuals with a documented or claimed history of cardiac conduction problems; individuals with severe vomiting or diarrhoea; and individuals with clinical malaria confirmed by an RDT.

Before enrolment, written informed consent was obtained from participants, who received a copy of the signed consent form. For those who could not read or write Khmer, a fingerprint was obtained and countersigned by an impartial witness. For participants aged 16–18 years, written informed assent was also obtained.

The trial was approved by the Cambodian National Health Research Ethics Committee (reference NECHR316), and by the University of Oxford Tropical Research Ethics Committee (reference 23-19). The study protocol has been published.^18^

### Randomisation and masking

Eligible participants were randomly assigned 1:1 to one of the two treatment groups according to a computer-generated randomisation schedule. Randomisation was in permuted blocks of size ten and stratified by village. Individual, sealed, and sequentially numbered opaque envelopes were provided for each trial site, with one envelope per participant indicating the treatment allocation. Allocation was done by trained study staff drawing the next envelope, which contained the study number and treatment allocation. An open-label design was selected due to unavailability of a suitable placebo that was identical in appearance to artemether–lumefantrine. Masking of investigators or participants was not possible. The randomisation procedure allowed for drug allocation concealment before envelopes were opened. All laboratory investigations were performed without knowledge of the treatment allocation.

### Procedures

Before initiation of the study, a series of community sensitisation and engagement activities were conducted in villages. Information focused on the concepts of chemoprophylaxis, malaria transmission, and exposure to mosquitos in forests. Engagement activities included meetings conducted by a dedicated community engagement team led by staff fluent in Khmer and local languages, supported by the district health department, administrative authorities, village malaria workers, and village leaders. Details of community engagement and acceptability have been published separately.^19^

At baseline, basic demographic and epidemiological data were collected. A physical examination and a symptom questionnaire were completed by qualified study staff. All prescribed medications used within the previous 7 days and a history of any drug allergies were recorded. An RDT for the detection of malaria was performed for participants with fever or a history of fever in the previous 24 h. Volunteers who tested positive were excluded and received standard antimalarial treatment from the village malaria worker.

Together with the National Center for Parasitology, Entomology and Malaria Control (Phnom Penh, Cambodia), artemether–lumefantrine was chosen as the study intervention, to be compared with a multivitamin (with no antimalarial effect) in the control group of the trial. Artemether–lumefantrine was obtained as Coartem from Novartis Saglik, Istanbul, Turkey (batch: KP663; manufacture date: August, 2019; expiry date: July, 2021). Each tablet contained 20 mg of artemether and 120 mg of lumefantrine. The multivitamin used in the trial was NANPROVIT-TAB, composed of vitamin A (retinol; 2500 IU), vitamin D3 (200 IU), vitamin B1 hydrochloride (thiamine; 5 mg), vitamin B2 (2 mg), vitamin B6 hydrochloride (pyridoxine; 2 mg), nicotinamide (4 mg), vitamin C (30 mg), and calcium pantothenate (1 mg), and was manufactured by Chea Chamnan Laboratoire, Phnom Penh, Cambodia (lot number: 200847). Participants received either two doses of artemether–lumefantrine (four tablets per dose) or two doses of multivitamin (one tablet per dose) per day for 3 days initially, followed by the same daily dosing once a week while travelling in the forest and for a further 4 weeks after leaving the forest. The first dose of study drugs was administered as directly observed therapy (DOT) by trained study staff. For this first dose, the full dose or a half dose of the study drug was repeated in case of vomiting within 30 min or 60 min, respectively. Participants were informed that the multivitamin had no antimalarial effect, and all were encouraged to continue to use other measures to protect themselves against malaria throughout. Subsequent doses were administered as so-called smart DOT, whereby intake was observed by a dedicated volunteer within the group of forest goers.

Participants were followed up between days 28 and 35 (1 month), between days 56 and 63 (2 months), and between days 84 and 91 (3 months)—ie, after each of three consecutive periods of 28 days plus a 7-day window. During follow-up, data were collected on duration of stay and locations visited in the forest, purpose of forest visits, accompanying forest goers, and potential risk factors for malaria infection. Brief physical examinations were performed, including aural temperature, and a symptom questionnaire was completed during each follow-up visit by qualified staff members. Participants were asked to report any diagnostic tests or treatment for malaria since the last follow-up visit. In case participants declared at the time of the follow-up visit no intention to return to the forest in the coming 4-week period, no further follow-up visits were scheduled, but 4 weeks of terminal chemoprophylaxis following their last day in the forest were completed. For those who declared an intention to return to the forest, they continued in the study for another period of 28 days up to a maximum of three periods.

Passive surveillance of clinical malaria continued throughout the study period, and records from local treatment providers were collected routinely. For participants who had an episode of RDT-confirmed clinical malaria at any time after enrolment up to the last follow-up visit, dried blood spots (three spots, 200 μL for each spot) for parasite DNA were collected on Whatman filter paper (Cytivia, Marlborough, MA, USA) by the village malaria worker. Laboratory procedures were the same as for baseline blood spot samples described below. Participants with malaria were treated by the village malaria worker in accordance with Cambodian malaria treatment guidelines (artesunate–mefloquine for both vivax and falciparum malaria).

Dried blood spots (three 200 μL spots) were collected on Whatman filter paper from finger pricks for parasite PCR from all participants at baseline, immediately before drug administration, and at each follow-up visit. Samples were stored in a plastic ziplock bag with silica gel and were sent to the Molecular Tropical Medicine Laboratory at Mahidol Oxford Tropical Medicine Research Unit, Bangkok, Thailand. Parasite DNA was extracted using the QIAmp DNA Mini Kit (Qiagen, Hilden, Germany) following the manufacturer's instructions. For *Plasmodium* identification, nested PCR targeting the 18S rRNA gene was performed as previously described; this method has a lower limit of detection of 1–10 parasites per μL.^20^ In brief, 1 μL of genomic DNA was used in a 20 μL reaction with outer primers rPLU1 and rPLU5. Then, nested PCR was performed with 2 μL of the primary PCR product and species-specific primers for the four human malaria species in separate reaction tubes. All PCR products were separated on 2% agarose gels. After staining with ethidium bromide, the gel was visualised under an ultraviolet light.

For assessment of study drug adherence in 43 randomly selected study participants who received artemether–lumefantrine, a 1 mL venous blood sample was collected in pre-chilled heparin tubes at the 4th, 8th, and 12th week follow-up visits for assessment of plasma lumefantrine concentrations. Allowing for anticipated loss to follow-up, this method would provide 100 samples for analysis. This testing was feasible only in villages in which pre-chilled heparin tubes could be used during follow-up. Randomisation was done by computer using the list of enrolled individuals in these villages. Samples were transported back on ice to the local laboratory for centrifugation at 2000 g for 7 min, after which 0·5 mL plasma was obtained and stored in liquid nitrogen. Plasma samples were sent on dry ice to the Pharmacology Laboratory at Mahidol Oxford Tropical Medicine Research Unit, Bangkok, Thailand, for measurement of lumefantrine concentrations using published methods.^21^

### Outcomes

The primary outcome was a composite endpoint of either clinical malaria with any *Plasmodium* species within 1–28, 29–56, or 57–84 days, or subclinical infection detected by PCR on days 28, 56, or 84. The first secondary outcome was the same as the primary outcome but for each species. Incidence of adverse events by study group as a measure of tolerability and safety and blood plasma concentrations of lumefantrine from 43 randomly selected individuals at each follow-up visit were additional prespecified secondary endpoints. Other secondary outcomes will be reported elsewhere separately and are listed in appendix 1 (p 12).

### Statistical analysis

It was anticipated that there would be a 5% *P falciparum* PCR positivity rate in the control group during each 28-day follow-up period. A total of 1605 person-months at risk (a month being 28 days) per group would be sufficient to detect a reduction in the PCR positivity rate of at least 40% with 80% power and 5% significance level—ie, 5% in those receiving multivitamin versus 3% in those receiving artemether–lumefantrine. To account for reduction in statistical power due to repeated observations in the same participant and loss to follow-up, we planned to include approximately 600 additional person-months at risk in each study group. Based on these considerations, the overall sample size was estimated to be 4400 person-months at risk (ie, 2200 person-months at risk in the treatment group and 2200 person-months at risk in the control group). Thus, with expected event rates of 5% in the control group versus 3% in the intervention group, we expected to observe 110 events in the control group and 66 events in the intervention group, giving a total of 176 events. The sample size calculations were performed in Stata IC version 15. The trial was stopped before the planned sample size was reached because enrolment and follow-up were slowed substantially by restrictions imposed due to the COVID-19 pandemic, and the grants that funded the trial came to an end.

The primary outcome was analysed by intention-to-treat, followed by a per-protocol analysis. Efficacy of artemether–lumefantrine versus multivitamin, here defined as the proportion of participants who remained uninfected over the 28-day episode, was summarised using proportions and 95% CIs. Crude proportions were calculated using the exact binomial 95% CIs. The absolute risk differences, protective efficacies, and risk ratios between artemether–lumefantrine and multivitamin were reported along with the corresponding 95% CIs. The robust standard errors were used to adjust for intracluster correlation of months at risk from the same individual (clustering within individual) using the generalised estimating equation approach with exchangeable correlation structure. Tests of significance were at the 5% level.

Best and worst case scenarios were initially planned to handle missing data.^18^ However, there is increasing literature that indicates that complete-case analysis performs similarly to multiple imputation in randomised controlled trials and sometimes even better than multiple imputation.^22^, ^23^, ^24^ Our assumption is that the outcome data in our study are likely to be missing completely at random and in this case, complete-case analysis would probably perform better than extreme case (best and worst case) analysis. There is an amendment section in the statistical analysis plan (appendix 2 p 16) with more details.

Adverse events were graded according to Common Terminology Criteria for Adverse Events version 5.0; November, 2017.^25^ All adverse event summaries referred to adverse events that newly started or increased in intensity after study drug administration. Adverse event summaries were generated for all adverse events that occurred after study drug administration, until the end of follow-up. Adverse events were reported for all participants by study group according to their incidence, intensity, and relationship to the study drug. Serious adverse events were reported separately. Statistical analysis was done using Stata MP version 16.

Monitoring was coordinated by the Clinical Trials Support Group within the Mahidol Oxford Tropical Medicine Research Unit. The trial is registered with ClinicalTrials.gov (NCT04041973) and is complete.

### Role of the funding source

The funder of the study had no role in study design, data collection, data analysis, data interpretation, or writing of the report.

## Results

**Figure 1:**
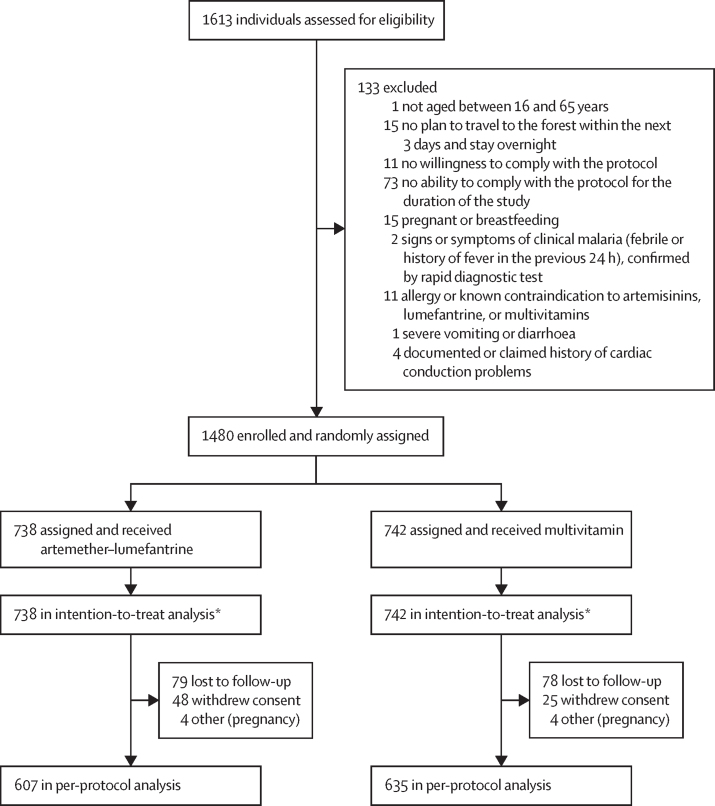
Trial profile *Within the intention-to-treat population, there were 713 complete cases in the artemether–lumefantrine group and 714 complete cases in the multivitamin group.

Between March 11 and Nov 20, 2020, a total of 1613 people were screened and 1480 enrolled (figure 1). The main reasons for exclusion (n=133) were not being able (n=73) or willing (n=11) to comply with the study protocol, no plan to travel to the forest within the next 3 days and stay overnight (n=15), and pregnancy or breastfeeding (n=15). Thus, 1480 participants received study drugs, 738 in the artemether–lumefantrine group and 742 in the multivitamin group. The two intervention groups were well balanced for all baseline characteristics (table 1). The median age was 30 years (IQR 21–40), 77% of participants were male, 96% were farmers, and 57% had previous malaria episodes. Symptoms at baseline were uncommon (120 [8%] of 1480 participants). Median time spent in the forest per participant episode was 17 nights (IQR 6–26) in each of the artemether–lumefantrine and multivitamin groups.

**Table 1:** Baseline characteristics

		**Artemether–lumefantrine (n=738)**	**Multivitamin (n=742)**	**Total (n=1480)**
**Demographics**				
Age, years		29 (21–40)	30 (21–40)	30 (21–40)
Sex				
	Male	546 (74%)	589 (79%)	1135 (77%)
	Female	192 (26%)	153 (21%)	345 (23%)
Weight, kg		52 (7)	52 (8)	52 (7)
Nationality				
	Khmer	737 (>99%)	740 (>99%)	1477 (>99%)
	Laos	1 (<1%)	2 (<1%)	3 (<1%)
Occupation				
	Farmer	706 (96%)	712 (96%)	1418 (96%)
	Student	10 (1%)	15 (2%)	25 (2%)
	Soldier	11 (1%)	8 (1%)	19 (1%)
	Teacher	5 (<1%)	3 (<1%)	8 (<1%)
	Housewife	3 (<1%)	0	3 (<1%)
	Fishing	1 (<1%)	1 (<1%)	2 (<1%)
	Village leader	1 (<1%)	1 (<1%)	2 (<1%)
	Village malaria worker	0	1 (<1%)	1 (<1%)
	Seller	0	1 (<1%)	1 (<1%)
	Environment department	1 (<1%)	0	1 (<1%)
Medical history*				
Previous malaria episodes		427 (58%)	421 (57%)	848 (57%)
Dengue fever		0	1 (<1%)	1 (<1%)
**Symptoms**				
Headache		30 (4%)	26 (4%)	56 (4%)
Dizziness		18 (2%)	20 (3%)	38 (3%)
Abdominal pain		9 (1%)	14 (2%)	23 (2%)
Joint pain		12 (2%)	6 (1%)	18 (1%)
Fatigue		4 (<1%)	4 (<1%)	8 (<1%)
Muscle pain		5 (<1%)	2 (<1%)	7 (<1%)
Diarrhoea		4 (<1%)	1 (<1%)	5 (<1%)
Itching or rash		1 (<1%)	2 (<1%)	3 (<1%)
Nausea		0	1 (<1%)	1 (<1%)
Vomiting		0	1 (<1%)	1 (<1%)
**Physical examination**				
Tympanic temperature†, °C		36·6 (0·4)	36·5 (0·4)	36·6 (0·4)
Movement abnormality		0	0	0
Skin abnormality		0	0	0
Eye abnormality		0	0	0
Breathing abnormality		0	0	0
Speech abnormality		0	0	0
Hearing abnormality		0	0	0

Data are median (IQR), n (%), or mean (SD).

* History within previous 5 years.

† At screening.

**Figure 2:**
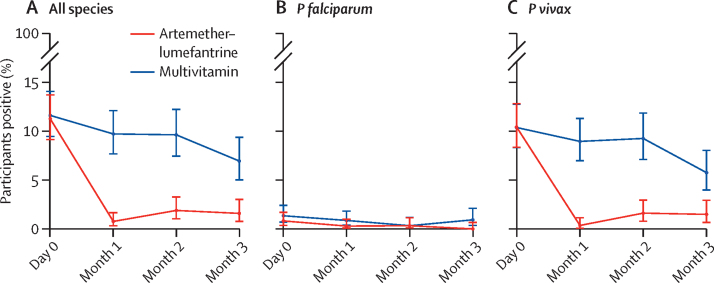
Percentage of participants with malaria infection over time Malaria infection was defined as PCR parasite positivity on days 0 (baseline), 28–35 (month 1), 56–63 (month 2), or 84–91 (month 3), or a case of confirmed clinical malaria during month 1, month 2, or month 3.

The proportion of participants who had parasitaemia detected by PCR at baseline was similar in both treatment groups (83 [11%] of 738 in the artemether–lumefantrine group and 86 [12%] of 742 in the multivitamin group). 713 participants in the artemether–lumefantrine group and 714 in the multivitamin group had a PCR result or confirmed clinical malaria by RDT during follow-up. Over the whole follow-up period, there were 14 episodes due to *P falciparum* infection (parasitaemia or clinical malaria), 166 due to *P vivax*, two due to *Plasmodium malariae*, and three due to mixed infections with both *P falciparum* and *P vivax* (figure 2 and appendix 1 pp 13–14). Combining all *Plasmodium* species, 19 (3%, 95% CI 2–4) of 713 participants had parasitaemia or clinical malaria in the artemether–lumefantrine group and 123 (17%, 15–20) of 714 in the multivitamin group; absolute risk difference 15% (95% CI 12–18; p<0·0001). This result translates to a protective efficacy of 85% (95% CI 75–90; p<0·0001) and a risk ratio of 0·15 (95% CI 0·1–0·25) in favour of artemether–lumefantrine (see appendix 1 p 5 for the intention-to-treat analysis and appendix 1 p 6 for the per-protocol analysis). The proportion of participants with parasitaemia or clinical malaria at 1, 2, or 3 months of follow-up was also lower in the artemether–lumefantrine group, with protective efficacies of 92% (95% CI 81–97) at 1 month, 81% (64–90) at 2 months, and 78% (52–89) at 3 months (appendix 1 p 5). Stratification of the results according to the *Plasmodium* PCR positivity status at day 0 showed that the efficacy of chemoprophylaxis with artemether–lumefantrine was higher in both groups overall and at all individual timepoints (appendix 1 pp 4–11).

The proportion of participants who had symptomatic or asymptomatic *P falciparum* infections during the follow-up period was lower with artemether–lumefantrine than with multivitamin: 0·3% (95% CI 0·03–1·01; two of 713) versus 1·7% (0·9–2·9; 12 of 714; absolute risk difference 1·4%, 95% CI 0·4–2·4, p=0·013; protective efficacy 83%, 95% CI 26–96, p=0·019; figure 2B; appendix 1 p 7). Fewer than ten participants in each group had a PCR-positive result for *P falciparum* on enrolment, hence an analysis for this subgroup was not meaningful; however, we have provided a summary for completeness in appendix 1 (p 6).

The proportion of participants who had symptomatic or asymptomatic *P vivax* infections was lower in the artemether–lumefantrine than the multivitamin group (figure 2C; appendix 1 pp 8–11) for all follow-up months combined: 18 (3%, 95% CI 2–4) of 713 participants in the artemether–lumefantrine group versus 112 (16%, 13–19) of 714 in the multivitamin group (absolute risk difference 13%, 95% CI 10–16, p<0·0001; protective efficacy 84%, 95% CI 74–90, p<0·0001). When stratified by the presence of *P vivax* parasitaemia at baseline, the artemether–lumefantrine chemoprophylactic effect for developing *P vivax* infection was numerically larger for participants without parasitaemia at baseline: protective efficacy for all months was 86% (74–92, p<0·0001) in PCR-negative participants and 80% (57–90; p<0·0001) in PCR-positive participants (appendix 1 p 8). In addition to the higher incidence, there was also longer persistence of *P vivax* infections (post-hoc analysis) in the multivitamin group compared with the artemether–lumefantrine group (appendix 1 pp 2, 12). Participants in the multivitamin group could have been infected and therefore potentially transmitting *P vivax* for 69 person-months (69 of 1819 person-months at risk in total) compared with 10 person-months (10 of 1796) in the artemether–lumefantrine group (incidence rate ratio 6·8, 95% CI 3·5–14·8, p<0·0001; appendix 1 p 12).

Overall loss to follow-up during months 1, 2, and 3, respectively, was 5% (39 of 738), 7% (44 of 629), and 8% (48 of 569) in the artemether–lumefantrine group and 5% (35 of 742), 8% (53 of 649), and 7% (41 of 581) in the multivitamin group (appendix 1 pp 15–16). Overall reported adherence to the full course of medication during each follow-up period was 97% (95% CI 96–98; 1797 completed courses out of 1854 courses started) in the artemether–lumefantrine group and 98% (97–98; 1842 completed courses out of 1885 courses started) in the multivitamin group (appendix 1 p 17). There were no significant differences in any of the measures of adherence between the artemether–lumefantrine group and the multivitamin group (appendix 1 p 17).

There were no significant differences between the artemether–lumefantrine group and multivitamin group in vomiting within 1 h (table 2). Although rare, there were higher rates of abdominal pain, fatigue, muscle pain, diarrhoea, and loss of appetite in the artemether–lumefantrine group than the multivitamin group, with overall prevalences of adverse events of 1·9% (355 events in 18 806 doses) in the artemether–lumefantrine group and 1·1% (207 events in 19 132 doses) in the multivitamin group (p<0·0001; table 2). No serious adverse events were reported as related to study drugs. There were two serious adverse events in the artemether–lumefantrine group and three in the multivitamin group. These events comprised one episode of dengue fever and one episode of hypoglycaemia in the artemether–lumefantrine group, and one suspected hepatocellular carcinoma and two accidents in the multivitamin group (table 2, appendix 1 pp 18–19).

**Table 2:** Treatment course uptake and adverse events

		**Artemether–lumefantrine**	**Multivitamin**	p value*
Number of participants		738	742	..
Number of participants participating in courses				
	Course 1 (all participants)	738	742	..
	Course 2 (total attended follow-up visit 1 plus total rejoined second follow-up period)	715	721	..
	Course 3 (total attended follow-up visit 2 plus total rejoined third follow-up period)	611	621	..
Number of participants who took more than one dose†		716	721	..
Number of doses taken		18 806	19 132	..
Vomiting within 1 h‡		1 (<0·1%)	0	0·50
Adverse events‡				
	Headache	71 (0·4%)	51 (0·3%)	0·070
	Abdominal pain	52 (0·3%)	24 (0·1%)	0·0012
	Dizziness	45 (0·2%)	32 (0·2%)	0·14
	Fatigue	34 (0·2%)	17 (<0·1%)	0·017
	Joint pain	28 (0·1%)	16 (<0·1%)	0·071
	Muscle pain	23 (0·1%)	11 (<0·1%)	0·040
	Diarrhoea	19 (0·1%)	8 (<0·1%)	0·035
	Fever	15 (<0·1%)	7 (<0·1%)	0·092
	Loss of appetite	15 (<0·1%)	4 (<0·1%)	0·012
	Cough	14 (<0·1%)	11 (<0·1%)	0·56
	Sore throat	11 (<0·1%)	8 (<0·1%)	0·50
	Nausea	7 (<0·1%)	4 (<0·1%)	0·39
	Itching	3 (<0·1%)	2 (<0·1%)	0·69
	Vomiting	3 (<0·1%)	1 (<0·1%)	0·37
	Others	15 (<0·1%)	11 (<0·1%)	0·44
	All adverse events	355 (1·9%)	207 (1·1%)	<0·0001
Serious adverse events‡§		2 (<0·1%)	3 (<0·1%)	1·00

Data are n, n/N (%), or n (%).

* Comparing between groups using Fisher's exact test.

† All participants (738 in the artemether–lumefantrine group, 742 in the multivitamin group) took the first dose after enrolment; for 22 and 21 participants, respectively, it was not recorded that they took any further doses.

‡ The denominator for the percentages is the total number of doses taken in each group (18 806 in the artemether–lumefantrine group, 19 312 in the multivitamin group).

§ Serious adverse events included one case of dengue fever and one case of hypoglycaemia in the artemether–lumefantrine group, and one suspected hepatocellular carcinoma and two accidents in the multivitamin group.

Median time from the last dose of artemether–lumefantrine to blood sampling for lumefantrine assessment was 1 day (IQR 1–2) for those with detectable plasma lumefantrine and 1 day (1·00–1·75) for those with undetectable plasma lumefantrine. Of 105 blood samples taken from 43 randomly selected participants from three villages in the artemether–lumefantrine group at follow-up (40 first, 37 second, and 28 third follow-up visits), 99 (94%) were positive for lumefantrine and 83 (79%) for desbutyl-lumefantrine. Five of the six participants who had no detectable plasma lumefantrine or desbutyl-lumefantrine said they had taken the artemether–lumefantrine in the correct dose with food as per the schedule, and intake had been observed by the forest team leader (smart DOT). The remaining participant said they had missed the last dose. Of the 105 participants with a drug measurement, none had a confirmed clinical episode of malaria during follow-up, and none of the six participants with undetectable lumefantrine and five (5%) of the 99 participants with detectable drug concentrations were positive by PCR for *P vivax*.

## Discussion

The trial found that it is feasible and effective to give forest goers antimalarial chemoprophylaxis with artemether–lumefantrine. Antimalarial chemoprophylaxis with artemether–lumefantrine had a large impact on the proportions of participants with subsequent parasitaemia: 3% among those who received chemoprophylaxis compared with 17% in the control group. A marked impact on parasitaemia occurred irrespective of whether participants were PCR-positive on enrolment. The large differences in outcome between the treatment groups strongly suggest that the forest goers adhered to their respective regimens, in concordance with the other measures of adherence reported. Adverse events reported by trial participants receiving artemether–lumefantrine and multivitamin were similar, reinforcing the well known safety and tolerability of artemether–lumefantrine.

Most infections were with *P vivax*, and a large proportion of these were probably caused by relapses from pre-existing liver hypnozoites, rather than new infections. Relapses of vivax malaria are not prevented by a blood schizontocidal drug such as artemether–lumefantrine but can be suppressed by artemether–lumefantrine, and the findings do suggest a major impact on persistent *P vivax* carriage. Participants in the control group were found to carry *P vivax* infections for 69 person-months compared with 10 person-months in the artemether–lumefantrine group. This more than six-fold difference might have provided a degree of indirect protection for forest goers in the control group who accompanied those receiving artemether–lumefantrine chemoprophylaxis and could have contributed to the decrease in parasite prevalence over time observed in the control group. A potential indirect protective effect of artemether–lumefantrine on the control group might therefore have resulted in slightly underestimating the prophylactic protective effect of artemether–lumefantrine. The study detected only 11 clinical malaria episodes during the study period (0·4% of participants in the artemether–lumefantrine group and 1% in the control group), suggesting a similar impact on clinical malaria as on parasitaemia.

The findings come at a time when forested regions have become some of the last remaining foci of malaria transmission in southeast Asia. A range of interventions to interrupt malaria transmission has been tried with mixed success in the Greater Mekong subregion.^5^ Some interventions such as mosquito-proof hammocks are cumbersome and have been shown to have low efficacy,^9^ while others, including mass screening and treatment, have yet to show benefit.^26^ The use of antimalarial chemoprophylaxis for high-risk groups is not a new concept.^27^ Seasonal malaria chemoprevention targets children growing up in the Sahel countries, where the transmission intensity is in general much higher than in the Greater Mekong subregion.^28^, ^29^ Yet the parasite prevalence in forest goers in this study of over 10% at baseline is relatively high. Seasonal malaria chemoprevention is popular with the target population and has been found to be highly cost-effective.^19^ More than 10 million children living in the Sahel now receive seasonal malaria chemoprevention annually. Due to the fundamental differences in malaria epidemiology between the Sahel and Greater Mekong subregion, adult forest goers and not children are at highest risk in the Greater Mekong subregion. Other than in the military, this study is the first time that widescale antimalarial chemoprophylaxis has been targeted at high-risk groups in the Greater Mekong subregion. It is generally recommended that different antimalarials are used for first-line malaria treatment and for mass treatment to reduce the risk of developing drug resistance. In Cambodia, the first-line antimalarial treatment is artesunate–mefloquine. When deploying artemether–lumefantrine in the context of chemoprophylaxis, adherence is very important because with adherence to a full 3-day course the risk of the parasite developing resistance is not greater than with usual malaria treatment since the window for selection is the same.^30^

The study was conducted in 2020 during the first year of the COVID-19 pandemic, which made access to study sites difficult. Repeated lockdowns prohibited large gatherings, including sensitisation meetings with forest goers, and required unusual flexibility from all members of the study team throughout the study period. The well balanced baseline characteristics of both study groups indicate that the randomisation was achieved successfully despite the constraints. Restrictions due to COVID-19 slowed enrolment and follow-up, and thus the trial had to be stopped before reaching the planned sample size once the grants came to an end. Despite this, we believe the statistically significant and approximately six-fold difference between study groups in subsequent infections is sufficiently reliable evidence from which to report a real effect of the intervention. The COVID-19 pandemic had no noticeable negative impact on malaria control in Cambodia, despite valid concerns that diagnosis and treatment would be undermined. Quite the opposite has occurred, and clinical malaria has reached unprecedented low levels in Cambodia, while numbers of malaria diagnostic tests performed suggests this observation is not due to under-reporting. Our study detected only 11 clinical malaria cases, which was insufficient to detect statistically significant differences between study groups.

Given the nature of forest work, DOT could not be provided by a trained health worker, and instead smart DOT was used, whereby the first dose was observed by a health worker and subsequent doses supervised by a volunteer forest worker in each group. This meant the study was conducted closer to a real-world setting. Despite this, high participant adherence was achieved, which is supported by the finding that the large majority of malaria cases occurred in the control group.

The antimalarial regimen used in this study for chemoprophylaxis did not include 8-aminoquinolines required for the prevention of *P vivax* relapse, without which the interruption of transmission of *P vivax* becomes an extended process. Given the increasing relative importance of vivax malaria in the Greater Mekong subregion, future studies should consider the addition of 8-aminoquinolines, such as primaquine or tafenoquine.^31^ Tafenoquine would provide both radical cure of *P vivax* and causal prophylaxis of *P falciparum* infections, but will require highly reliable screening for glucose-6-phosphate dehydrogenase (G6PD) deficiency of forest goers, because of the risk of haemolysis. With a new generation of point-of-care diagnostic tools for G6PD deficiency approaching regulatory approval, such a strategy might become feasible in the future.^32^ A combination of ACT and tafenoquine chemoprophylaxis should be considered for elimination of both species, particularly where *P vivax* predominates.

Limitations of artemether–lumefantrine are the relatively short duration of the prophylactic effect compared with other ACTs, the dependence on concomitant intake of a fat, and the twice daily dosing. Advantages of artemether–lumefantrine for chemoprophylaxis in Cambodia are the antimalarial efficacy, that it is not used as first-line antimalarial treatment, and the very good safety and tolerability profile. Good tolerability and an excellent safety profile were especially important as the participants receiving it would be otherwise healthy and thus potentially less likely to accept drug-related symptoms than patients with clinical malaria. In other studies,^33^, ^34^ artemether–lumefantrine has been associated with mild adverse drug reactions—chiefly, headache, dizziness, weakness, muscle or joint pain, and tiredness. We also observed a small, self-reported increase of these symptoms in the present study in those taking artemether–lumefantrine. The study shows that, as anticipated, artemether–lumefantrine has a benign tolerability profile and in this regard is suitable for chemoprophylaxis. If other drugs are selected for chemoprophylaxis, it will be important that they are also reasonably well tolerated, or adherence might fall below the high levels observed in the present study. Because of the urgency of using this strategy, both Cambodia and Laos have recently implemented chemoprophylaxis in forest goers (artesunate–mefloquine in Cambodia and artesunate–pyronaridine in Laos).

In conclusion, this study demonstrated that chemoprophylaxis with artemether–lumefantrine was acceptable, well tolerated, and reduced by approximately six-fold the number of subsequent malaria infections over a 3-month period. Malaria chemoprophylaxis among high-risk groups such as forest goers is a valuable additional tool for malaria elimination in the Greater Mekong subregion.

## Data sharing

Deidentified, individual participant data that underlie this Article, along with a data dictionary describing variables in the dataset, are available to researchers whose proposed purpose of use is approved by the Mahidol University Oxford Tropical Medicine Research Unit data access committee. Related documents such as the study protocol and informed consent form will be made available on request. To request the dataset, please send a signed data request form to datasharing@tropmedres.ac. The data request form can be found online at https://www.tropmedres.ac/units/moru-bangkok/bioethics-engagement/data-sharing.

## Declaration of interests

We declare no competing interests.
