# Supplementary material for: Antimalarial chemoprophylaxis for forest goers in southeast Asia: an open-label, individually randomised controlled trial
Source: Lancet Infect Dis. 2023 Jan;23(1):81–90. doi: 10.1016/S1473-3099(22)00492-3 (PMC9763125; doi:10.1016/S1473-3099(22)00492-3)
Supplement: Supplementary appendix 1 [file mmc1.pdf]

THE LANCET
Infectious Diseases

Supplementary appendix 1

This appendix formed part of the original submission and has been peer reviewed. We post it as supplied by the authors.

Supplement to: Tripura R, von Seidlein L, Sovannaroth S, et al. Antimalarial chemoprophylaxis for forest goers in southeast Asia: an open-label, individually randomised controlled trial. *Lancet Infect Dis* 2022; published online Sept 26. https://doi.org/10.1016/S1473-3099(22)00492-3.

Supplementary Information

Secondary outcomes

1. A composite endpoint of either clinical malaria within 1-28, 29-56 or 57-84 days, or subclinical infection detected by PCR on days 28, 56 or 84 for each species.

Secondary outcomes 2 to 8 will be reported elsewhere:

2. Quantification of the impact of the ACT AL as prophylaxis for forest goers on overall malaria transmission using mathematical modelling.

3. Assessment of the impact of AL prophylaxis on the spread of genetic markers of artemisinin (such as Kelch13 mutations) and partner drug resistance.

4. Data on the place of residence, work, recent travel history and risk behaviours of forest goers in order to improve the understanding of high-risk groups, locations of malaria transmission and possible routes spread of malaria and artemisinin resistance.

5. Duration, location and purpose of individual forest visits.

6. Detailed data and Global Positioning System (GPS) mapping on a subset of participants and their peers relating to the behaviours and risk factors associated with malaria infection in order to improve understanding of local malaria transmission among forest goers.

7. Prevalence of asymptomatic Plasmodium infections in high-risk populations at varying seasonal time points.

8. Prevalence of other infectious diseases that affect the study population.

Figure S1. Percentage of participants with malaria infection over time by PCR status at baseline. Malaria infection was defined as all species PCR parasite positivity on days 0 (baseline), 28-35 (month 1), 56-63 (month 2) or 84-91 (month 3) or a case of confirmed clinical malaria during days month 1, month 2 or month 3. All species: A. PCR negative at baseline, B. PCR positive at baseline. *P. vivax*: C. PCR negative at baseline, D. PCR positive at baseline. *P. falciparum*: E. PCR negative at baseline, F. PCR positive at baseline.

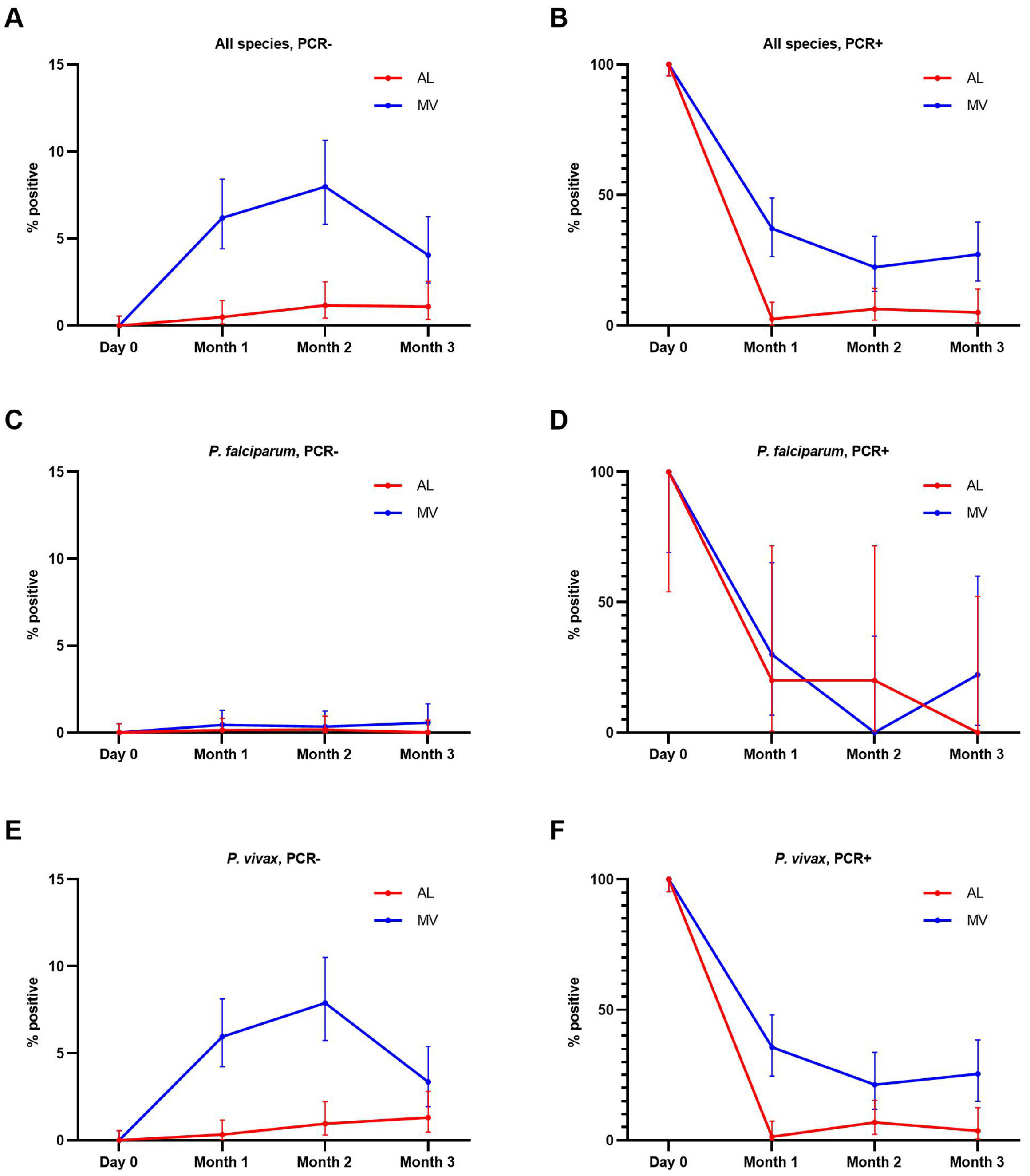

Figure S2. Comparison of incidence and persistence of *P.vivax* infections between the AL (artemether-lumefantrine) and the MV (Multivitamin) arms.

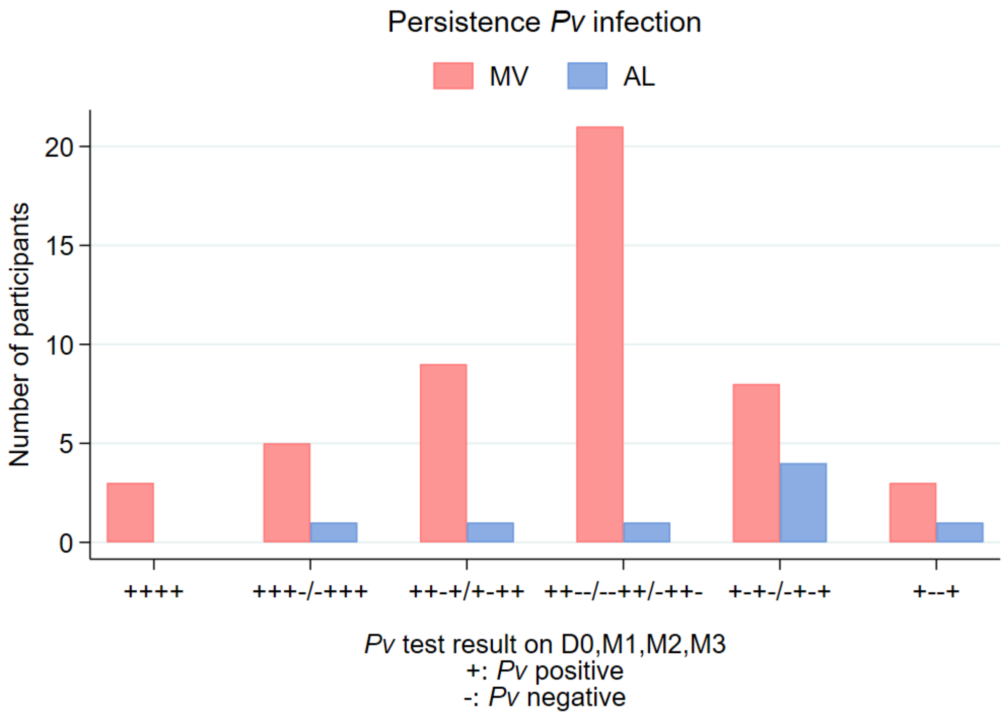

Table S1. 28-day PCR positivity or confirmed clinical malaria of any species by study arm.

	All participants (N=1,480)		Participants with PCR negative at D0 (N=1,311)		Participants with PCR positive at D0 (N=169: 11 *Pf*, 5 *Pf+Pv*, 4 *Pm*, 148 *Pv*, 1 *Pv+Pm*)	
	AL	MV	AL	MV	AL	MV
Number of participants	738	742	655	656	83	86
**Composite endpoint (PCR positivity or confirmed clinical malaria), n/N (%; 95% CI)** [a]						
Any months	19/713 (3; 2 – 4)	123/714 (17; 15 – 20)	11/632 (2; 0.9 – 3)	82/634 (13; 10 – 16)	8/81 (10; 4 – 19)	41/80 (51; 40 – 63)
Month 1[*]	5/687 (0.7; 0.2 – 2)	67/691 (10; 8 – 12)	3/609 (0.5; 0.2 – 1)	38/613 (6; 4 – 8)	2/78 (3; 0.3 – 9)	29/78 (37; 27 – 49)
Month 2[*]	11/594 (2; 0.9 – 3)	57/593 (10; 8 – 12)	6/516 (1; 0.4 – 3)	42/526 (8; 6 – 11)	5/78 (6; 2 – 14)	15/67 (22; 13 – 34)
Month 3[*]	8/515 (2; 0.7 – 3)	37/535 (7; 5 – 9)	5/455 (1; 0.4 – 3)	19/469 (4; 2 – 6)	3/60 (5; 1 – 14)	18/66 (27; 17 – 40)
Number of episodes from all months [c]	24/1,796 (1; 0.7 – 2)	161/1,819 (9; 7 – 10)	14/1,580 (0.9; 0.3 – 1)	99/1,608 (6; 5 – 7)	10/216 (5; 1 – 8)	62/211 (29; 22 – 37)
**1) 28-day PCR positivity, n/N (%)** [a,b]						
Any months	16/713 (2)	116/714 (16)	8/632 (1)	78/634 (12)	8/81 (10)	38/80 (48)
Month 1[*]	5/687 (0.7)	64/691 (9)	3/609 (0.5)	38/613 (6)	2/78 (3)	26/78 (33)
Month 2[*]	8/594 (1)	54/593 (9)	3/516 (0.6)	39/526 (7)	5/78 (6)	15/67 (22)
Month 3[*]	8/515 (2)	35/535 (7)	5/455 (1)	17/469 (4)	3/60 (5)	18/66 (27)
Number of episodes from all months	21/1,796 (1)	153/1,819 (8)	11/1,580 (0.7)	94/1,608 (6)	10/216 (5)	59/211 (28)
**2) Confirmed clinical malaria (during follow-up), n/N (%)** [a, **]						
Any months	3/713 (0.4)	8/714 (1)	3/632 (0.5)	5/634 (0.8)	0/81 (0)	3/80 (4)
Month 1	0/687 (0)	3/691 (0.4)	0/609 (0)	0/613 (0)	0/78 (0)	3/78 (4)
Month 2	3/594 (0.5)	3/593 (0.5)	3/516 (0.6)	3/526 (0.6)	0/78 (0)	0/67 (0)
Month 3	0/515 (0)	2/535 (0.4)	0/455 (0)	2/469 (0.4)	0/60 (0)	0/66 (0)
Number of episodes from all months	3/1,796 (0.2)	8/1,819 (0.4)	3/1,580 (0.2)	5/1,608 (0.3)	0/216 (0)	3/211 (1)

[a] *Denominators are the numbers of participants who had a PCR result (±1 day of the scheduled FU period) or confirmed clinical malaria result.*
[b] *Included PCR positive result without confirmed clinical malaria.*
[c] *Adjusted for clustering*
[*] *Included participants who had PCR result at one day before and after the schedule period.*
[**] *There were 2 confirmed clinical malaria cases detected after day 84 but within 120 days after screening (on D96 and D116) that are not in this table (both PV confirmed by RDT).*

Table S2a. ITT Analysis: Protective efficacy of the ACT artemether-lumefantrine versus a multivitamin preparation as defined by the 28-day PCR parasite positivity or confirmed clinical malaria for all species.

	Efficacy rate, n/N (%; 95% CI) [a]		AL protective efficacy (95% CI)	p-value
	AL	Multivitamin		
**All participants**	**(N=738)**	**(N=742)**		
All months	694/713 (97; 96 – 98)	591/714 (83; 80 – 85)	85 (75 – 90)	<0.0001
Month 1	682/687 (99; 98 – 100)	624/691 (90; 88 – 92)	92 (81 – 97)	<0.0001
Month 2	583/594 (98; 97 – 99)	536/593 (90; 88 – 93)	81 (64 – 90)	<0.0001
Month 3	507/515 (98; 97 – 99)	498/535 (93; 91 – 95)	78 (52 – 89)	<0.0001
Individuals treated as 28-day - episodes[b]	1,772/1,796 (99; 98 – 99)	1,658/1,819 (91; 90 – 93)	85 (74 – 91)	<0.0001
**Participants with PCR negative at D0**	**(N=655)**	**(N=656)**		
All months	621/632 (98; 97 – 99)	552/634 (87; 84 – 90)	87 (75 – 93)	<0.0001
Month 1	606/609 (100; 99 – 100)	575/613 (94; 92 – 96)	92 (74 – 98)	<0.0001
Month 2	510/516 (99; 97 – 100)	484/526 (92; 89 – 94)	85 (66 – 94)	<0.0001
Month 3	450/455 (99; 97 – 100)	450/469 (96; 94 – 98)	73 (28 – 90)	0.0088
Individuals treated as 28-day - episodes[b]	1,566/1,580 (99; 99 – 100)	1,509/1,608 (94; 93 – 95)	86 (71 – 93)	<0.0001
**Participants with PCR positive at D0**	**(N=83)**	**(N=86)**		
All months	73/81 (90; 81 – 96)	39/80 (49; 37 – 60)	81 (62 – 90)	<0.0001
Month 1	76/78 (97; 91 – 100)	49/78 (63; 51 – 74)	93 (72 – 98)	<0.0001
Month 2	73/78 (94; 86 – 98)	52/67 (78; 66 – 87)	71 (25 – 89)	0.011
Month 3	57/60 (95; 86 – 99)	48/66 (73; 60 – 83)	82 (41 – 94)	0.0045
Individuals treated as 28-day - episodes[b]	206/216 (95; 92 – 99)	149/211 (71; 63 – 78)	84 (65 – 93)	<0.0001

[a] *Denominators are the numbers of participants who had a PCR result (±1 day of the scheduled follow-up period) or confirmed clinical malaria result.*
[b] *Adjusted for clustering*

Table S2b. Per Protocol Analysis: Protective efficacy of the ACT artemether-lumefantrine versus a multivitamin preparation as defined by the 28-day PCR parasite positivity or confirmed clinical malaria for <u>all species</u>

	Efficacy rate, n/N (%; 95% CI) [a]		AL protective efficacy (95% CI)	p-value
	AL	Multivitamin		
**All participants**	**(N=607)**	**(N=635)**		
All months	588/605 (97; 96 – 98)	515/630 (82; 79 – 85)	85 (75 – 91)	<0.0001
Month 1	579/584 (99; 98 – 100)	548/609 (90; 87 – 92)	91 (79 – 97)	<0.0001
Month 2	539/548 (98; 97 – 99)	507/562 (90; 87 – 93)	83 (66 – 92)	<0.0001
Month 3	505/513 (98; 97 – 99)	498/535 (93; 91 – 95)	77 (52 – 89)	0.0001
Individuals treated as 28-day - episodes[b]	1,623/1,645 (99; 98 – 99)	1,553/1,706 (91; 89 – 93)	85 (74 – 91)	<0.0001
**Participants with PCR negative at D0**	**(N=540)**	**(N=558)**		
All months	528/538 (98; 97 – 99)	479/554 (86; 83 – 89)	86 (74 – 93)	<0.0001
Month 1	516/519 (99; 98 – 100)	502/535 (94; 91 – 96)	91 (70 – 97)	0.0001
Month 2	476/481 (99; 98 – 100)	456/496 (92; 89 – 94)	87 (68 – 95)	<0.0001
Month 3	448/453 (99; 97 – 100)	450/469 (96; 94 – 98)	73 (28 – 90)	0.0091
Individuals treated as 28-day - episodes[b]	1,440/1,453 (99; 98 – 100)	1,408/1,500 (94; 92 – 95)	85 (70 – 93)	<0.0001
**Participants with PCR positive at D0**	**(N=67)**	**(N=77)**		
All months	60/67 (90; 80 – 96)	36/76 (47; 36 – 59)	80 (59 – 90)	<0.0001
Month 1	63/65 (97; 89 – 100)	46/74 (62; 50 – 73)	92 (67 – 98)	0.0004
Month 2	63/67 (94; 85 – 98)	51/66 (77; 65 – 87)	74 (25 – 91)	0.013
Month 3	57/60 (95; 86 – 99)	48/66 (73; 60 – 83)	82 (41 – 94)	0.0045
Individuals treated as 28-day - episodes[b]	183/192 (95; 90 – 98)	145/206 (70; 62 – 77)	84 (63 – 93)	<0.0001

[a] *Denominators are the numbers of participants who had a PCR result (±1 day of the scheduled follow-up period) or confirmed clinical malaria result.*
[b] *Adjusted for clustering*

Table S3. Protective efficacy of the ACT artemether-lumefantrine versus a multivitamin preparation as defined by the 28-day PCR parasite positivity or confirmed clinical malaria for *P. falciparum.*

	Efficacy rate, n/N (%; 95% CI) [a]		AL protective efficacy (95% CI)	p-value
	AL	Multivitamin		
**All participants**	**(N=738)**	**(N=742)**		
All months	711/713 (100; 99 – 100)	702/714 (98; 97 – 99)	83 (26 – 96)	0.019
Month 1	685/687 (100; 99 – 100)	685/691 (99; 98 – 100)	66 (0 – 93)	0.18
Month 2	592/594 (100; 99 – 100)	591/593 (100; 99 – 100)	0 (0 – 86)	1.00
Month 3	515/515 (100; 99 – 100)	530/535 (99; 98 – 100)	100 (NA)	NA
Individuals treated as 28-day - episodes[b]	1,792/1,796 (100; 99 – 100)	1,806/1,819 (99; 99 – 100)	69 (0 – 93)	0.13
**Participants with PCR *Pf* negative at D0**	**(N=732)**	**(N=732)**		
All months	707/708 (100; 99 – 100)	697/704 (99; 98 – 100)	86 (0 – 98)	0.068
Month 1	681/682 (100; 99 – 100)	678/681 (100; 99 – 100)	67 (0 – 97)	0.34
Month 2	588/589 (100; 99 – 100)	583/585 (100; 99 – 100)	50 (0 – 95)	0.57
Month 3	510/510 (100; 99 – 100)	523/526 (99; 98 – 100)	100 (NA)	NA
Individuals treated as 28-day - episodes[b]	1,779/1,781 (100; 100 – 100)	1,784/1792 (100; 99 – 100)	75 (0 – 97)	0.20
**Participants with PCR *Pf* positive at D0** [c,d]	**(N=6)**	**(N=10)**		
All months	4/5 (80; 28 – 99)	5/10 (50; 19 – 81)	60 (0 – 94)	0.33
Month 1	4/5 (80; 28 – 99)	7/10 (70; 35 – 93)	33 (0 – 91)	0.69
Month 2	4/5 (80; 28 – 99)	8/8 (100; 63 – 100)	NA	NA
Month 3	5/5 (100; 48 – 100)	7/9 (78; 40 – 97)	100 (NA)	NA
Individuals treated as 28-day - episodes[b]	13/15 (87; 61 – 100)	22/27 (81; 70 – 93)	28 (0 – 89)	0.74

[a] *Denominators are the numbers of participants who had PCR result (±1 day of the scheduled FU period) or confirmed clinical malaria result.*
[b] *Adjusted for clustering*
[c] *Included Pf+Pv*
[d] *Numbers were very low (less than 10 in each group) that had a Pf PCR positive result on day 0, hence no meaningful summaries are obtained.*
NA: Protective efficacy and/or confidence intervals for the protective efficacy cannot be calculated.

Table S4. Protective efficacy of the ACT artemether-lumefantrine versus a multivitamin preparation as defined by the 28-day PCR parasite positivity or confirmed clinical malaria for P. vivax.

	Efficacy rate, n/N (%; 95% CI) [a]		AL protective efficacy (95% CI)	p-value
	AL	Multivitamin		
**All participants**	**(N=738)**	**(N=742)**		
All months	695/713 (97; 96 – 98)	602/714 (84; 81 – 87)	84 (74 – 90)	<0.0001
Month 1	684/687 (100; 99 – 100)	629/691 (91; 89 – 93)	95 (85 – 98)	<0.0001
Month 2	584/594 (98; 97 – 99)	538/593 (91; 88 – 93)	82 (65 – 91)	<0.0001
Month 3	507/515 (98; 97 – 99)	504/535 (94; 92 – 96)	73 (42 – 88)	0.0008
Individuals treated as 28-day - episodes[b]	1,775/1,796 (99; 98 – 99)	1,671/1,819 (92; 90 – 93)	86 (76 – 92)	<0.0001
**Participants with PCR *Pv* negative at D0**	**(N=661)**	**(N=665)**		
All months	626/637 (98; 97 – 99)	563/642 (88; 85 – 90)	86 (74 – 92)	<0.0001
Month 1	612/614 (100; 99 – 100)	584/621 (94; 92 – 96)	95 (77 – 99)	0.0001
Month 2	516/521 (99; 98 – 100)	490/532 (92; 89 – 94)	88 (70 – 95)	<0.0001
Month 3	454/460 (99; 97 – 100)	460/476 (97; 95 – 98)	61 (2 – 85)	0.046
Individuals treated as 28-day - episodes[b]	1,582/1,595 (99; 99 – 100)	1,534/1,629 (94; 93 – 95)	86 (72 – 93)	<0.0001
**Participants with PCR *Pv* positive at D0** [c]	**(N=77)**	**(N=77)**		
All months	69/76 (91; 82 – 96)	39/72 (54; 42 – 66)	80 (57 – 90)	<0.0001
Month 1	72/73 (99; 93 – 100)	45/70 (64; 52 – 75)	96 (72 – 99)	0.0012
Month 2	68/73 (93; 85 – 98)	48/61 (79; 66 – 88)	68 (15 – 88)	0.022
Month 3	53/55 (96; 87 – 100)	44/59 (75; 62 – 85)	86 (40 – 97)	0.0076
Individuals treated as 28-day - episodes[b]	193/201 (96; 93 – 99)	137/190 (72; 64 – 80)	86 (68 – 94)	<0.0001

[a] *Denominators are the number of participants who had PCR result (±1 day of the scheduled FU period) or confirmed clinical malaria result.*

[b] *Adjusted for clustering*

[c] *Included Pf+Pv and Pm+Pv*

Table S5. 28-day PCR positivity or confirmed clinical malaria of *P. vivax*.

	All participants (N=1,480)		Participants with PCR *Pv* negative at D0 (N=1,326)		Participants with PCR *Pv* positive at D0 (N=154 *Pv* Positive: 148 *Pv*, 5 *Pf+Pv,* 1 *Pm+Pv*)	
	AL	MV	AL	MV	AL	MV
Number of participants	738	742	661	665	77	77
**Composite endpoint (PCR *Pv* positivity or confirmed clinical malaria), n/N (%; 95% CI)** [a]						
Any months	18/713 (3; 2 – 4)	112/714 (16; 13 – 19)	11/637 (1.7; 0.9 – 3)	79/642 (12; 10 – 15)	7/76 (9; 4 – 18)	33/72 (46; 34 – 58)
Month 1[*]	3/687 (0.4; 0.1 – 1)	62/691 (9; 7 – 11)	2/614 (0.3; 0.04 – 1)	37/621 (6; 4 – 8)	1/73 (1; 0.03 – 7)	25/70 (36; 25 – 48)
Month 2[*]	10/594 (2; 0.8 – 3)	55/593 (9; 7 – 12)	5/521 (1; 0.3 – 2)	42/532 (8; 6 – 11)	5/73 (7; 2 – 15)	13/61 (21; 12 – 34)
Month 3[*]	8/515 (2; 0.7 – 3)	31/535 (6; 4 – 8)	6/460 (1; 0.5 – 3)	16/476 (3; 2 – 5)	2/55 (4; 0.4 – 13)	15/59 (25; 15 – 38)
Number of episodes from all months [c]	21/1,796 (1; 0.6 – 2)	148/1,819 (8; 7 – 10)	13/1,595 (0.8; 0.3 – 1)	95/1,629 (6; 5 – 7)	8/201 (4; 1 – 7)	53/190 (28; 20 – 36)
**1) 28-day PCR *Pv* positivity, n/N (%)** [a,b]						
Any months	15/713 (2)	106/714 (15)	8/637 (1)	76/642 (12)	7/76 (9)	30/72 (42)
Month 1[*]	3/687 (0.4)	59/691 (9)	2/614 (0.3)	37/621 (6)	1/73 (1)	22/70 (31)
Month 2[*]	7/594 (1)	53/593 (9)	2/521 (0.4)	40/532 (8)	5/73 (7)	13/61 (21)
Month 3[*]	8/515 (2)	29/535 (5)	6/460 (1)	14/476 (3)	2/55 (4)	15/59 (25)
Number of episodes from all months	18/1,796 (1)	141/1,819 (8)	10/1,595 (0.6)	91/1,629 (6)	8/201 (4)	50/190 (26)
**2) Confirmed *Pv* clinical malaria (between day 0 – day 28), n/N (%)** [a, **]						
Any months	3/713 (0.4)	7/714 (1)	3/637 (0.5)	4/642 (0.6)	0/76 (0)	3/72 (4)
Month 1	0/687 (0)	3/691 (0.4)	0/614 (0)	0/621 (0)	0/73 (0)	3/70 (4)
Month 2	3/594 (0.5)	2/593 (0.3)	3/521 (0.6)	2/532 (0.4)	0/73 (0)	0/61 (0)

Month 3	0/515 (0)	2/535 (0.4)	0/460 (0)	2/476 (0.4)	0/55 (0)	0/59 (0)
Number of episodes from all months	3/1,796 (0.2)	7/1,819 (0.4)	3/1,595 (0.2)	4/1,629 (0.2)	0/201 (0)	3/190 (2)

[a] *Denominators are the number of participants who had a PCR result (±1 day of the scheduled follow-up period) or confirmed clinical malaria result.*

[b] *Included PCR Pv positive result without confirmed clinical malaria.*

[c] *Adjusted for clustering*

[*] *Included participants who had PCR result at one day before and after the schedule period.*

[**] *There were 2 confirmed clinical malaria cases detected after day 84 but within 120 days after screening (on D96 and D116) that are not in this table (both PV confirmed by RDT).*

Table S6. Efficacy of the ACT artemether-lumefantrine versus a multivitamin preparation as defined by the 28-day PCR parasite positivity or confirmed clinical malaria for *P. vivax*.

	Efficacy rate, n/N (%; 95% CI) [a]		Risks difference (95% CI)	p-value
	AL	Multivitamin		
**All participants**	**(N=738)**	**(N=742)**		
All months	695/713 (97; 96 – 98)	602/714 (84; 81 – 87)	13.2 (10.3 – 16.1)	<0.0001
Month 1	684/687 (100; 99 – 100)	629/691 (91; 89 – 93)	8.5 (6.3 – 10.7)	<0.0001
Month 2	584/594 (98; 97 – 99)	538/593 (91; 88 – 93)	7.6 (5.0 – 10.1)	<0.0001
Month 3	507/515 (98; 97 – 99)	504/535 (94; 92 – 96)	4.2 (2.0 – 6.5)	0.0002
Individuals treated as 28-day - episodes[b]	1,775/1,796 (99; 98 – 99)	1,671/1,819 (92; 90 – 93)	7.0 (5.4 – 8.6)	<0.0001
**Participants with PCR *Pv* negative at D0**	**(N=661)**	**(N=665)**		
All months	626/637 (98; 97 – 99)	563/642 (88; 85 – 90)	10.6 (7.8 – 13.3)	<0.0001
Month 1	612/614 (100; 99 – 100)	584/621 (94; 92 – 96)	5.6 (3.7 – 7.5)	<0.0001
Month 2	516/521 (99; 98 – 100)	490/532 (92; 89 – 94)	6.9 (4.5 – 9.4)	<0.0001
Month 3	454/460 (99; 97 – 100)	460/476 (97; 95 – 98)	2.1 (0.1 – 4.0)	0.036
Individuals treated as 28-day - episodes[b]	1,582/1,595 (99; 99 – 100)	1,534/1,629 (94; 93 – 95)	5.0 (3.6 – 6.4)	<0.0001
**Participants with PCR *Pv* positive at D0** [c]	**(N=77)**	**(N=77)**		
All months	69/76 (91; 82 – 96)	39/72 (54; 42 – 66)	36.6 (23.4 – 49.8)	<0.0001
Month 1	72/73 (99; 93 – 100)	45/70 (64; 52 – 75)	34.3 (22.8 – 45.9)	<0.0001
Month 2	68/73 (93; 85 – 98)	48/61 (79; 66 – 88)	14.5 (2.7 – 26.3)	0.016
Month 3	53/55 (96; 87 – 100)	44/59 (75; 62 – 85)	21.8 (9.6 – 33.9)	0.0004
Individuals treated as 28-day - episodes[b]	193/201 (96; 93 – 99)	137/190 (72; 64 – 80)	23.9 (15.3 – 32.6)	<0.0001

[a] *Denominators are the number of participants who had a PCR result (±1 day of the scheduled follow-up period) or confirmed clinical malaria result.*

[b] *Adjusted for clustering*

[c] *Included Pf+Pv and Pm+Pv*

Table S7. Incidence of *P. vivax* infections.

DOM1M2M3 pattern*	Numbers		Duration (months)	Person months	
	MV	AL		MV	AL
++++	3	0	3	9	0
+++- / -+++	5	1	2	10	2
++-+ / +-++	9	1	2	18	2
++-- / --++ / -++-	21	1	1	21	1
+-+- / -+-+	8	4	1	8	4
+--+	3	1	1	3	1
Total infections	49	8		69	10
Total observations	1,819	1,796			

*DO = baseline, M1 = 0-28 days, M2 = 29-56 days, M3 = 57-84 days.

We Assumed that the duration of a ++++ infection is at least 3 months, a +++-/-+++/++-+ / +-++ infection 2 months, and a ++--/-++-/--++ /+-+- / -+-+/ +--+ infection is at least one month. Based on this definition/assumption, we observed that participants in the MV arm could have been infected (and therefore potentially infectious for 69 person-months compared to 10 person-months in the AL arm and this was significant 69/1,819 vs 10/1,796, IRR 6.8, 95% CI (3.5, 14.8), p<0.0001.

Table S8. Numbers of each species of 28-day PCR positivity or confirmed clinical malaria.

Study arm	Time point	All participants/episodes [a]	Negative	Positive					
				Pf	*Pv*	*Pm*	*Pf+Pv*	*Pm+Pv*	All
**All participants**									
All	D0	1,480	1,311	11	148	4	5	1	169
	M1	1,378	1,306	6	63	1	2	0	72
	M2	1,187	1,119	3	64	0	1	0	68
	M3	1,050	1,005	5	39	1	0	0	45
	M1,2,3	3,615	3,430	14	166	2	3	0	185
AL	D0	738	655	4	74	2	2	1	83
	M1	687	682	2	3	0	0	0	5
	M2	594	583	1	9	0	1	0	11
	M3	515	507	0	8	0	0	0	8
	M1,2,3	1,796	1,772	3	20	0	1	0	24
MV	D0	742	656	7	74	2	3	0	86
	M1	691	624	4	60	1	2	0	67
	M2	593	536	2	55	0	0	0	57
	M3	535	498	5	31	1	0	0	37
	M1,2,3	1,819	1,658	11	146	2	2	0	161
**Participants with PCR-D0**									
All	D0	1,311	1,311	0	0	0	0	0	0
	M1	1,222	1,181	3	37	0	1	0	41
	M2	1,042	994	2	46	0	0	0	48
	M3	924	900	3	21	0	0	0	24
	M1,2,3	3,188	3,075	8	104	0	1	0	113
AL	D0	655	655	0	0	0	0	0	0
	M1	609	606	1	2	0	0	0	3
	M2	516	510	1	5	0	0	0	6
	M3	455	450	0	5	0	0	0	5
	M1,2,3	1,580	1,566	2	12	0	0	0	14
MV	D0	656	656	0	0	0	0	0	0
	M1	613	575	2	35	0	1	0	38
	M2	526	484	1	41	0	0	0	42
	M3	469	450	3	16	0	0	0	19
	M1,2,3	1,608	1,509	6	92	0	1	0	99
**Participants with PCR+ D0**									
All	D0	169	0	11	148	4	5	1	169
	M1	156	125	3	26	1	1	0	31

	M2	145	125	1	18	0	1	0	20
	M3	126	105	2	18	1	0	0	21
	M1,2,3	427	355	6	62	2	2	0	72
AL	D0	83	0	4	74	2	2	1	83
	M1	78	76	1	1	0	0	0	2
	M2	78	73	0	4	0	1	0	5
	M3	60	57	0	3	0	0	0	3
	M1,2,3	216	206	1	8	0	1	0	10
MV	D0	86	0	7	74	2	3	0	86
	M1	78	49	2	25	1	1	0	29
	M2	67	52	1	14	0	0	0	15
	M3	66	48	2	15	1	0	0	18
	M1,2,3	211	149	5	54	2	1	0	62

[a] *Included participants who had a PCR result (±1 day of the scheduled follow-up period) or confirmed clinical malaria result.*

Table S9. Adherence to study follow-up.

	n (%) or n/N (%)		p-value [c]
	AL	Multivitamin	
Number of participants	738	742	
**Follow up 1**			
Loss to follow up/withdraw during month 1/missed FU1	39/738 (5)	35/742 (5)	0.64
Scheduled visit FU1 (D28-D35)	680/738 (92)	686/742 (92)	0.85
Unscheduled visit FU1*	19/738 (3)	21/742 (3)	0.87
Total visited FU1 (scheduled+unscheduled)	**699/738 (95)**	**707/742 (95)**	**0.64**
**Registration for FU2**			
Registered for FU2	629/699 (90)	649/707 (92)	0.27
Not registered for FU2	70/699 (10)	58/707 (8)	0.27
**Follow up 2**			
**Registered participants**			
Loss to follow up/withdraw during month 2/ missed FU2	44/629 (7)	53/649 (8)	0.46
Scheduled visit FU2 (D56-D63)	569/629 (90)	577/649 (89)	0.41
Unscheduled visit FU2	16/629 (3)	19/649 (3)	0.73
Total visited FU2 as registered (scheduled+unscheduled)	585/629 (93)	596/649 (92)	0.46
Redecision to visit FU2 [a]			
Redecided to join scheduled visit	3/70 (4)	1/58 (2)	0.63
Redecided to join unscheduled visit	0/70 (0)	0/58 (0)	NA
Total redecided to visit FU2 (scheduled+unscheduled)	3/70 (4)	1/58 (2)	0.63
Total not redecided to visit FU2	67/70 (96)	57/58 (98)	0.63
Rejoining visit FU2			
Rejoined scheduled visit	14/39 (36)	7/35 (20)	0.20
Rejoined unscheduled visit	2/39 (5)	7/35 (20)	0.075
Total rejoined visit FU2 (scheduled+unscheduled)	16/39 (41)	14/35 (40)	1.00
Total not rejoined visit FU2	23/39 (59)	21/35 (60)	1.00
Total visited FU2 (scheduled+unscheduled)	**604/738 (82)**	**611/742 (82)**	**0.84**
Visited scheduled FU2 (D56-D63)	586/738 (79)	585/742 (79)	0.80
Visited unscheduled FU2**	18/738 (2)	26/742 (4)	0.28
**Registration for FU3**			
Registered for FU3	569/604 (94)	581/611 (95)	0.53
Not registered for FU3	35/604 (6)	30/611 (5)	0.53
**Follow up 3**			
**Registered participants**			
Loss to follow up/withdraw during month 3/ missed FU3	48/569 (8)	41/581 (7)	0.44
Scheduled visit FU3 (D84-D91)	475/569 (83)	499/581 (86)	0.29
Unscheduled visit FU3	46/569 (8)	41/581 (7)	0.58
Total visited FU3 as registered (scheduled+unscheduled)	521/569 (92)	540/581 (93)	0.44
Redecision to visit FU3 [a]			

Redecided to join scheduled visit	1/35 (3)	0/30 (0)	1.00
Redecided to join unscheduled visit	0/35 (0)	1/30 (3)	0.46
Total redecided to visit FU3 (scheduled+unscheduled)	1/35 (3)	1/30 (3)	1.00
Rejoining visit FU3 [b]			
Rejoined scheduled visit	3/134 (2)	7/131 (5)	0.21
Rejoined unscheduled visit	4/134 (3)	3/131 (2)	1.00
Total rejoined visit FU3 (scheduled+unscheduled)	7/134 (9)	10/131 (18)	0.46
Total visited FU3 (scheduled+unscheduled)	**529/738 (72)**	**551/742 (74)**	**0.27**
Visited scheduled FU3 (D84-D91)	479/738 (65)	506/742 (68)	0.19
Visited unscheduled FU3***	50/738 (7)	45/742 (6)	0.60

FU1 = first follow-up (month 1), FU2 = second follow-up (month 2), FU3 = third follow-up (month 3)

[a] *Participants who did not register for the next follow-up but then attended for the next follow-up.*
[b] *Considering participants who missed FU2 plus those who missed FU1 and did not rejoin FU2 plus those who did not registere to join FU3 (and did not redecide to join later).*
[c] *Comparisons between groups using Fisher's exact test.*
**Participants came early on D26-D27 or late on D36-D42 for FU1 (D28-D35). Of these, 7 and 5 participants came as unscheduled for FU1 but within ±1 day in the AL and MV arms, respectively.*
*** Participants came early on D53-D55 or late on D64-D73 for FU2 (D56-D63). Of these, 6 and 6 participants came as unscheduled for FU2 but within ±1 day in the AL and MV arms, respectively. 2 of 3 participants in the AL arm and 2 of 3 participants in the MV arm who had clinical malaria during month 2 did not come to FU2.*
**** Participants came early on D75-D83 or late on D92-D98. Of these, 36 and 28 participants came as unscheduled for FU3 but within ±1 day in the AL and MV arms, respectively. 1 of 2 participant in the MV arm had clinical malaria during month 3 and did not come to FU3.*

Table S10. Adherence to study drug.

	n (%) or n/N (%)		p-value [a]
	AL	Multivitamin	
Number of participants	738	742	
Participation in courses	(N=738)	(N=742)	
Participated course 1 (D0-D28)	716 (97)	721 (97)	0.88
Participated course 2 (D35-D56)	611 (83)	617 (83)	0.75
Participated course 3 (D63-D84)	527 (71)	547 (74)	0.50
Participation in full course			
Course 1 (D0-D28)	694/716 (97)	703/721 (98)	0.53
Course 2 (D35-D56)	584/611 (96)	601/617 (97)	0.089
Course 3 (D63-D84)	519/527 (98)	538/547 (98)	1.00
Participation by number of courses	(N=738)	(N=742)	
No courses	22 (3)	21 (3)	0.88
Course 1 only	105 (14)	102 (14)	0.82
Courses 1 and 2 only	84 (11)	72 (10)	0.31
Courses 1 and 3 only	0 (0)	2 (0.3)	0.50
Courses 1 and 2 and 3	527 (71)	545 (73)	0.38

[a] *Comparing between groups using Fisher's exact test.*

Table S11. Signs and symptoms adverse events by severity.

Adverse events [a]	AL					Multivitamin				
	Highest grade					Highest grade				
	Mild	Moderate	Severe	Life-threatening	Fatal	Mild	Moderate	Severe	Life-threatening	Fatal
Headache	69 (97)	2 (3)	0 (0)	0 (0)	0	51 (100)	0 (0)	0 (0)	0 (0)	0 (0)
Abdominal pain	51 (98)	1 (2)	0 (0)	0 (0)	0 (0)	24 (100)	0 (0)	0 (0)	0 (0)	0 (0)
Dizziness	41 (91)	4 (9)	0 (0)	0 (0)	0 (0)	32 (100)	0 (0)	0 (0)	0 (0)	0 (0)
Fatigue	33 (97)	1 (3)	0 (0)	0 (0)	0 (0)	17 (100)	0 (0)	0 (0)	0 (0)	0 (0)
Joint pain	27 (96)	1 (4)	0 (0)	0 (0)	0 (0)	16 (100)	0 (0)	0 (0)	0 (0)	0 (0)
Muscle pain	22 (96)	1 (4)	0 (0)	0 (0)	0 (0)	11 (100)	0 (0)	0 (0)	0 (0)	0 (0)
Diarrhoea	18 (95)	1 (5)	0 (0)	0 (0)	0 (0)	8 (100)	0 (0)	0 (0)	0 (0)	0 (0)
Fever	15 (100)	0 (0)	0 (0)	0 (0)	0 (0)	7 (100)	0 (0)	0 (0)	0 (0)	0 (0)
Loss of appetite	14 (93)	1 (7)	0 (0)	0 (0)	0 (0)	4 (100)	0 (0)	0 (0)	0 (0)	0 (0)
Cough	14 (100)	0 (0)	0 (0)	0 (0)	0 (0)	11 (100)	0 (0)	0 (0)	0 (0)	0 (0)
Sore throat	11 (100)	0 (0)	0 (0)	0 (0)	0 (0)	8 (100)	0 (0)	0 (0)	0 (0)	0 (0)
Nausea	7 (100)	0 (0)	0 (0)	0 (0)	0 (0)	4 (100)	0 (0)	0 (0)	0 (0)	0 (0)
Itching	3 (100)	0 (0)	0 (0)	0 (0)	0 (0)	2 (100)	0 (0)	0 (0)	0 (0)	0 (0)
Vomiting	3 (100)	0 (0)	0 (0)	0 (0)	0 (0)	1 (100)	0 (0)	0 (0)	0 (0)	0 (0)
Others	15 (100)	0 (0)	0 (0)	0 (0)	0 (0)	9 (82)	2 (18)	0 (0)	0 (0)	0 (0)
**All adverse events**	343 (97)	12 (3)	0 (0)	0 (0)	0 (0)	205 (99)	2 (1)	0 (0)	0 (0)	0 (0)

[a] *Values in cells are n (%). The denominators for this table are number of AEs for each sign/symptom (row percentage).*

Table S12. Signs and symptoms adverse events by relationship to study drug.

	AL				Multivitamin			
	Relationship to drug				Relationship to drug			
Adverse events [a]	Unrelated	Probably	Possibly	Definitely	Unrelated	Probably	Possibly	Definitely
Headache	61 (86)	2 (3)	8 (11)	0 (0)	50 (98)	0 (0)	1 (2)	0 (0)
Abdominal pain	45 (87)	0 (0)	7 (13)	0 (0)	23 (96)	0 (0)	1 (4)	0 (0)
Dizziness	35 (78)	0 (0)	10 (22)	0 (0)	29 (91)	0 (0)	3 (9)	0 (0)
Fatigue	29 (85)	0 (0)	5 (15)	0 (0)	16 (94)	0 (0)	1 (6)	0 (0)
Joint pain	25 (89)	1 (4)	2 (7)	0 (0)	14 (88)	0 (0)	2 (13)	0 (0)
Muscle pain	21 (91)	0 (0)	2 (9)	0 (0)	10 (91)	0 (0)	1 (9)	0 (0)
Diarrhoea	18 (95)	0 (0)	1 (5)	0 (0)	8 (100)	0 (0)	0 (0)	0 (0)
Fever	14 (93)	0 (0)	1 (7)	0 (0)	7 (100)	0 (0)	0 (0)	0 (0)
Loss of appetite	14 (93)	0 (0)	1 (7)	0 (0)	3 (75)	0 (0)	1 (25)	0 (0)
Cough	14 (100)	0 (0)	0 (0)	0 (0)	11 (100)	0 (0)	0 (0)	0 (0)
Sore throat	11 (100)	0 (0)	0 (0)	0 (0)	7 (88)	0 (0)	1 (13)	0 (0)
Nausea	6 (86)	0 (0)	1 (14)	0 (0)	3 (75)	0 (0)	1 (25)	0 (0)
Itching	3 (100)	0 (0)	0 (0)	0 (0)	2 (100)	0 (0)	0 (0)	0 (0)
Vomiting	2 (67)	0 (0)	1 (33)	0 (0)	1 (100)	0 (0)	0 (0)	0 (0)
Others	15 (100)	0 (0)	0 (0)	0 (0)	10 (91)	1 (9)	0 (0)	0 (0)
**All adverse events**	313 (88)	3 (1)	39 (11)	0 (0)	194 (94)	1 (0)	12 (6)	0 (0)

[a] *Values in cells are n (%). The denominators for this table are numbers of AEs for each sign/symptom (row percentage).*
