# Supplementary material for: Antimalarial chemoprophylaxis for forest goers in southeast Asia: an open-label, individually randomised controlled trial
Source: Lancet Infect Dis. 2023 Jan;23(1):81–90. doi: 10.1016/S1473-3099(22)00492-3 (PMC9763125; doi:10.1016/S1473-3099(22)00492-3)
Supplement: Supplementary appendix 2 [file mmc2.pdf]

THE LANCET
Infectious Diseases

Supplementary appendix 2

This appendix formed part of the original submission and has been peer reviewed. We post it as supplied by the authors.

Supplement to: Tripura R, von Seidlein L, Sovannaroth S, et al. Antimalarial chemoprophylaxis for forest goers in southeast Asia: an open-label, individually randomised controlled trial. *Lancet Infect Dis* 2022; published online Sept 26. https://doi.org/10.1016/S1473-3099(22)00492-3.

STATISTICAL ANALYSIS PLAN

An open-label individually randomised controlled trial to assess the efficacy of artemether-lumefantrine prophylaxis for malaria among forest goers in Cambodia
Short title: Study to assess efficacy of artemether-lumefantrine prophylaxis against forest malaria in Cambodia

ACRONYM: PAL-Cambodia
Protocol no.: MAL19001

Version 1.0
31st March 2021

WRITTEN BY:

PIMNARA PEERAWARANUN

31/03/2021

Print name & signature | Date
Statistician

REVIEWED AND APPROVED BY:

PROF MAVUTO MUKAKA

31/03/2021

Print Name & signature | Date
Head of Statistics

PROF LORENZ VON SEIDLEIN

31/03/2021

Print Name & signature | Date
Investigator

PROF RICHARD MAUDE

31/03/2021

Print Name & signature | Date
Principal Investigator

TABLE OF CONTENTS

1. INTRODUCTION 3

2. STUDY OBJECTIVES AND ENDPOINTS 3

2.1 PRIMARY OBJECTIVE 3
2.2 PRIMARY ENDPOINT 3
2.3 SECONDARY OBJECTIVES AND ENDPOINTS 3
2.4 GENERAL 4
2.5 DETERMINATION OF SAMPLE SIZE 5

3. ANALYSIS CONSIDERATIONS 5

3.1 GENERAL ANALYSIS APPROACH 5
3.2 DATA INTEGRITY 5
3.3 DATA CLEANING AND VERIFICATION 5
3.4 LOCKING THE DATASET 6
3.5 DATA FORMAT AND ANALYSIS LOGS 6
3.6 INTERIM ANALYSES 6

4. DATA ANALYSIS 6

4.1 TRIAL PROFILE 6
4.2 DEMOGRAPHICS AND OTHER BASELINE CHARACTERISTICS 7
4.3 ANALYSIS OF PRIMARY OUTCOME 9
4.3.1 Handling of missing data 10
4.3.2 Comparison of the efficacy of the ACT artemether-lumefantrine versus a multivitamin preparation as defined by the 28-day, 56-day, 84-day PCR parasite positivity rate or incidence of confirmed clinical malaria of any species 10
4.4 ANALYSIS OF SECONDARY OUTCOMES 11
4.4.1 Comparison of the efficacy of the ACT artemether-lumefantrine versus a multivitamin preparation As defined by the 28-day PCR parasite positivity or confirmed clinical malaria for each species 11
4.4.2 Quantifying the impact of the ACT artemether-lumefantrine (AL) as prophylaxis for forest goers on overall malaria transmission using mathematical modelling. 11
4.4.3 Assessing the impact of artemether-lumefantrine prophylaxis on the spread of genetic markers of artemisinin (such as Kelch13 mutations) and partner drug resistance. 12
4.4.4 Details of place of residence, work, recent travel history and risk behaviours of forest goers in order to improve the understanding of high risk groups, locations of malaria transmission and possible routes spread of malaria and artemisinin resistance. 12
4.4.5 Adverse events and serious adverse events by study arms during the course of prophylaxis. 13
4.4.6 Prevalence of asymptomatic Plasmodium infections in high risk populations at varying seasonal time points 15

1. INTRODUCTION

In the Greater Mekong Subregion (GMS) adults are at highest risk for malaria. The most relevant disease vectors bite during daytime and outdoors which makes forest work a high-risk activity for malaria. The absence of effective vector control strategies and limited periods of exposure during forest visits suggest that chemoprophylaxis could be an appropriate strategy to protect forest workers against malaria. We propose to evaluate the feasibility and protective efficacy of antimalarial prophylaxis during forest work.

The proposed study will help to assess the efficacy and feasibility of prophylaxis to prevent malaria in forest workers, help to identify the optimal regimen, and predict its efficacy in reducing overall transmission. The proposed study is a critical step for future use of chemoprophylaxis to protect forest workers in the GMS against malaria.

This document sets out the statistical analysis plan for an open-label individually randomised controlled trial to assess the efficacy of artemether-lumefantrine (AL) prophylaxis for malaria among forest goers in Cambodia.

2. STUDY OBJECTIVES AND ENDPOINTS

2.1 PRIMARY OBJECTIVE

To compare the efficacy of the ACT artemether-lumefantrine versus a multivitamin preparation as defined by the 28-day **PCR parasite positivity rate or incidence of confirmed clinical malaria** of any species.

2.2 PRIMARY ENDPOINT

- The 28-day **PCR Plasmodium positivity rate** of any species or
- Proportion of participants with **confirmed clinical malaria** of any species reported between day 0 and day 28, at any and each of the Month 1, Month 2 or 3 follow-ups.

2.3 SECONDARY OBJECTIVES AND ENDPOINTS

This section outlines the secondary objectives and the corresponding endpoints. A detailed plan for analysis of some of these endpoints will be produced the specialised groups as shown below:

- To compare the efficacy of the ACT artemether-lumefantrine (AL) versus a multivitamin preparation as defined by the 28-day, 56-day and 84-day PCR parasite positivity rate or incidence of confirmed clinical malaria for each species.
  Composite endpoint
  - 28-day, 56-day and 84-day PCR Plasmodium positivity rate for each Plasmodium species or
  - Proportion of participants with confirmed malaria reported between day 0 and day 28 for each species

- To quantify the impact of the ACT artemether-lumefantrine (AL) as prophylaxis for forest goers on overall malaria transmission using mathematical modelling.

- To assess the impact of artemether-lumefantrine prophylaxis (AL) on the spread of genetic markers of artemisinin (such as Kelch13 mutations) and partner drug resistance.
  Endpoint
  - Prevalence of Kelch13 mutations and other genetic markers of antimalarial drug resistance of known functional significance

- To obtain data on the place of residence, work, recent travel history and risk behaviours of forest goers in order to improve the understanding of high risk groups, locations of malaria transmission and possible routes of spread of malaria and artemisinin resistance.
  Endpoint
  - Data on the place of residence, work, recent travel history and mobile phone use events covering the whole population during the course of prophylaxis.

- To obtain detailed data and GPS mapping on a subset of participants and their peers relating to the behaviours and risk factors associated with malaria infection in order to improve understanding of local malaria transmission among forest goers.
  Endpoint
  - Detailed data and GPS mapping on a subset of participants and their peers relating to the behaviours and risk factors associated with malaria infection

- To determine the prevalence of asymptomatic Plasmodium infections in high risk populations at varying seasonal time points.
  Endpoint
  - Overall prevalence of Plasmodium at baseline, stratified by season and risk factors

2.4 GENERAL

The study takes place at up to 50 villages in selected malaria endemic districts in Stung Treng and Pursat Provinces, Cambodia. As the malaria situation in these areas is dynamic, the villages will be identified prior to the start of the trial from analysis of up to date malaria incidence from passive surveillance collected by the Cambodia National Center for Parasitology Entomology and Malaria Control. If there are found to be insufficient villages with malaria cases in Stung Treng Province at the time of trial commencement, we will include additional sites in Pursat Province. MORU has existing study sites and teams in both provinces which will support the running of this trial. The rationale for choosing these areas include high forest cover and ongoing malaria transmission among forest goers.

2.5 DETERMINATION OF SAMPLE SIZE

The target population for this study will be adult Cambodians who work and sleep in the forest (farmers, collect forest goods, hunting, etc.). 2,200 study participant episodes are required in each arm to have sufficient power to detect a statistically significant difference between the treatment arm and a control arm. The estimate of the required sample size is complicated by the scarce data on *P.falciparum* (*Pf*) incidence in forest workers. Formally, we anticipate that the risk of being *Pf* positive without receiving prophylaxis will be around 5%. A total of 1,605 participant-episodes per arm are enough to detect a difference of at least 40% in the proportion of episodes with a *Pf* positive result as defined by the 28-day PCR parasite positivity rate i.e. from 5% positivity in participants without receiving antimalarial prophylaxis (i.e. multivitamin) to 3% positivity in participants receiving artemether-lumefantrine prophylaxis. This has been estimated with 80% power and 5% significance level. However, we also anticipate that we will likely observe multiple episodes being recruited into the study that can reduce power of the study if not accounted for. To compensate for the multiple episodes and any losses to follow up, we plan to recruit approximately 600 (i.e. 595) additional episodes in each group on top of the required 1605 single episodes. This gives an additional 27% episodes to account for the multiple episodes and losses to follow up. Thus, the overall sample size will be 4,400 episodes (i.e. 2,200 episodes in the treatment arm and 2,200 episodes in the control arm).The sample size calculations have been performed in Stata version 15.

3. ANALYSIS CONSIDERATIONS

3.1 GENERAL ANALYSIS APPROACH

The main analysis strategy for the primary outcome will be the intention-to-treat (ITT) principle. In this analysis, participants will be analysed according to the arm of randomisation irrespective of the treatment that was actually received.
These analyses will be followed by the per protocol (PP) analysis. In this analysis, only those that adhered to the protocol with respect to the primary outcome will be included for analysis of the primary outcome.
For some objectives, data analysis will mainly be performed using Stata 16 or higher, StataCorp, 4905 Lakeway Drive College Station, Texas 77845 USA. While R or Python will be used for other endpoints. Specialised analyses such as GPS mapping, and spatial analysis will use ArcGIS 10.3 or higher, ESRI, 380 New York Street, Redlands, California USA.

3.2 DATA INTEGRITY

This study will be conducted in compliance with the protocol, relevant Standard Operating Procedures (SOPs), Good Clinical Practice (GCP) and the applicable regulatory requirement(s). All the analyses will be performed on clean data only.

3.3 DATA CLEANING AND VERIFICATION

All data will be cleaned and verified prior to statistical analysis. The study site will be visited by the Monitor periodically at times agreed on with the Investigator. At the time of each monitoring visit, the Monitor will review the completed CRFs to ascertain that all items have been completed and that the data provided are accurate and obtained in the manner specified in the protocol. The Monitor will also check that the data in

the CRF are consistent with the clinical records (Source Data Verification [SDV]) and that study results are recorded completely and correctly. The data manager will ensure that clean data is submitted to the statistician for analysis. The statistician will cross-check that the available data for analysis is clean. Any data cleaning queries will need to be resolved before statistical analyses.

3.4 LOCKING THE DATASET

After data cleaning and responding to all data queries, the clean data will be locked normally in the database that was used for data capturing. The data may also be locked and stored in other user-friendly formats such as MS Excel and Stata. The locked data will be stored at an identifiable secure place and should be available to the relevant researchers upon request following proper request procedures. The data will also be in other backup media such as CDs or tapes.

3.5 DATA FORMAT AND ANALYSIS LOGS

Prior to dispensing data to the trial statistician, the head of data management will make sure that the data to be sent to the trial statistician is clean. This will help the statistician to provide the analysis results in a timely manner as there will be a reduced amount of queries if clean data is provided to the trial statistician. Data will be given to the Trial Statistician by the head of Data Management (or designated person) in a format that is compatible with statistical software reading. Statistical analyses will be performed in Stata, version 16 or higher. Statistical programs and output logs will be kept for all analysis and made available upon request.

3.6 INTERIM ANALYSES

There was no interim analysis for this trial. Hence, no adjustment will be made to account for interim analysis of the trial efficacy differences.

4. DATA ANALYSIS

4.1 TRIAL PROFILE

The number of participants who will be screened, reasons for non-enrolment, number of participants randomized, number of participants lost to follow up and the number of participants assessed for 28-day endpoint will be summarised in a CONSORT flow diagram, figure 1, below.

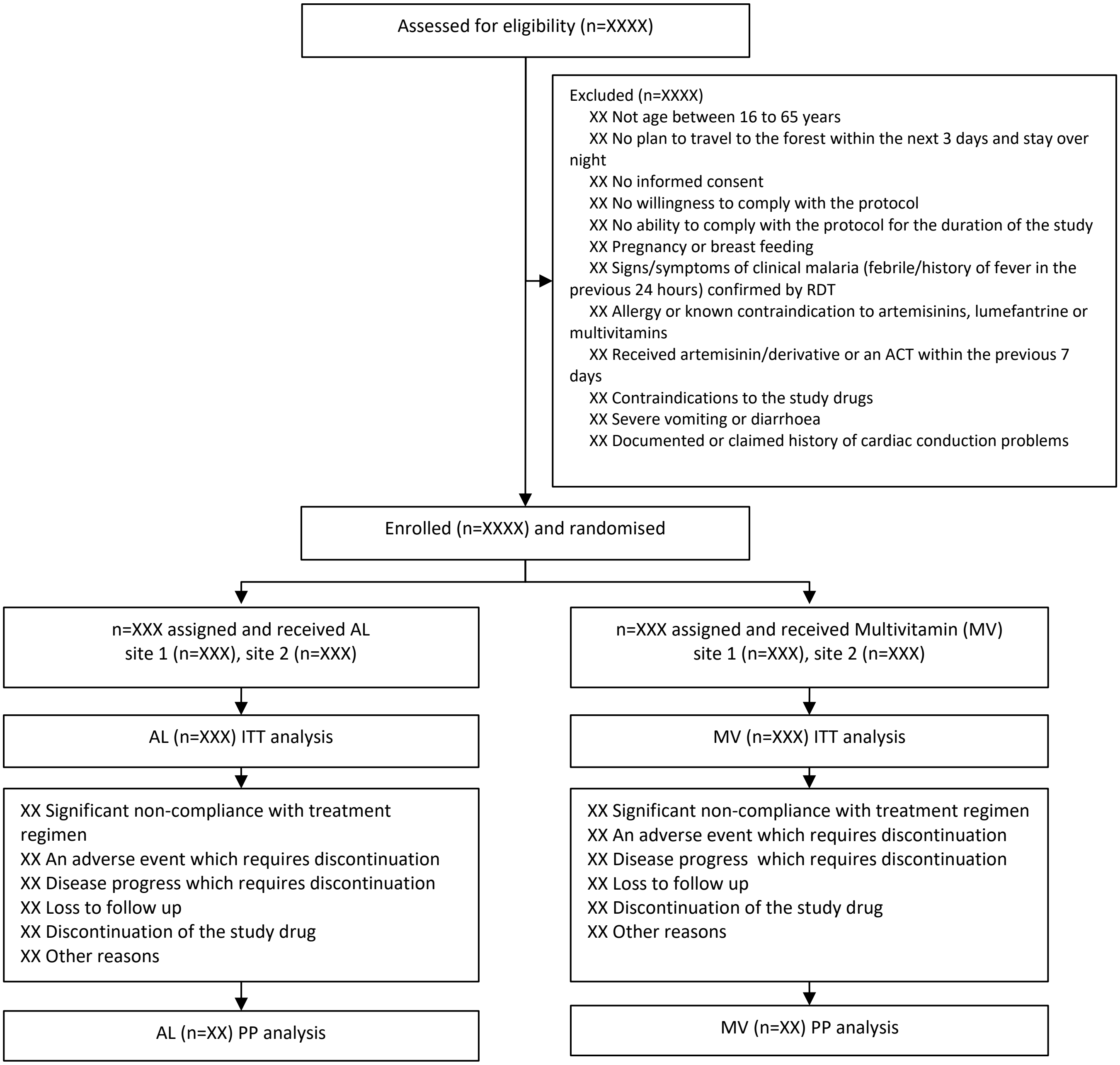

Figure 1 Consort Trial Profile by Arms

4.2 DEMOGRAPHICS AND OTHER BASELINE CHARACTERISTICS

The following baseline characteristics will be described by study arm in table 1 (below). Age will be summarized using median and interquartile range. Weight and tympanic temperature will be summarized using mean and standard deviation. Categorical variables such as sex and presence of symptoms at baseline will be summarized using frequencies and percentages.

Table 1. Baseline Characteristics of the Participants

Characteristics	Artemether-Lumefantrine (N=XXX)	Multivitamin (N=XXX)	Total (N=XXX)
**Demographics**			
Age (years), median (IQR)	XX.X (XX.X - XX.X)	XX.X (XX.X - XX.X)	XX.X (XX.X - XX.X)
Male sex, n (%)	XX (XX.X)	XX (XX.X)	XX (XX.X)
Weight (kg), mean (SD)	XX.X (XX.X)	XX.X (XX.X)	XX.X (XX.X)
Tympanic temperature* (°C), mean (SD)	XX.X (XX.X)	XX.X (XX.X)	XX.X (XX.X)
Nationality, n (%)			
Khmer	XX (XX.X)	XX (XX.X)	XX (XX.X)
Laos	XX (XX.X)	XX (XX.X)	XX (XX.X)
Thai	XX (XX.X)	XX (XX.X)	XX (XX.X)
Others (including English)	XX (XX.X)	XX (XX.X)	XX (XX.X)
Occupation, n (%)			
Farmer	XX (XX.X)	XX (XX.X)	XX (XX.X)
Fishing	XX (XX.X)	XX (XX.X)	XX (XX.X)
Student	XX (XX.X)	XX (XX.X)	XX (XX.X)
Millitary	XX (XX.X)	XX (XX.X)	XX (XX.X)
Housewife	XX (XX.X)	XX (XX.X)	XX (XX.X)
Others	XX (XX.X)	XX (XX.X)	XX (XX.X)
Place of residence, n (%)			
Stung Treng	XX (XX.X)	XX (XX.X)	XX (XX.X)
Siem Pang	XX (XX.X)	XX (XX.X)	XX (XX.X)
Others	XX (XX.X)	XX (XX.X)	XX (XX.X)
Participants with prior malaria episodes , n (%)	XX (XX.X)	XX (XX.X)	XX (XX.X)
**Symptoms**, n/N (%)			
Headache	XX/XX (XX.X)	XX/XX (XX.X)	XX (XX.X)
Loss of appetite	XX/XX (XX.X)	XX/XX (XX.X)	XX (XX.X)
Dizziness	XX/XX (XX.X)	XX/XX (XX.X)	XX (XX.X)
Joint pain	XX/XX (XX.X)	XX/XX (XX.X)	XX (XX.X)
Abdominal pain	XX/XX (XX.X)	XX/XX (XX.X)	XX (XX.X)
Fatigue	XX/XX (XX.X)	XX/XX (XX.X)	XX (XX.X)
Nausea	XX/XX (XX.X)	XX/XX (XX.X)	XX (XX.X)
Vomiting	XX/XX (XX.X)	XX/XX (XX.X)	XX (XX.X)
Diarrhoea	XX/XX (XX.X)	XX/XX (XX.X)	XX (XX.X)
Itching/ rash	XX/XX (XX.X)	XX/XX (XX.X)	XX (XX.X)

Cough	XX/XX (XX.X)	XX/XX (XX.X)	XX (XX.X)
**Antimalarial drug history**, n (%)			
Drug1	XX (XX.X)	XX (XX.X)	XX (XX.X)
Drug2	XX (XX.X)	XX (XX.X)	XX (XX.X)
Drug3	XX (XX.X)	XX (XX.X)	XX (XX.X)
ect.	XX (XX.X)	XX (XX.X)	XX (XX.X)
**Medical history**, n (%)			
Disease1	XX (XX.X)	XX (XX.X)	XX (XX.X)
Disease2	XX (XX.X)	XX (XX.X)	XX (XX.X)
Disease3	XX (XX.X)	XX (XX.X)	XX (XX.X)
etc.	XX (XX.X)	XX (XX.X)	XX (XX.X)
**Malaria history**, n (%)			
Malaria1	XX (XX.X)	XX (XX.X)	XX (XX.X)
Malaria1	XX (XX.X)	XX (XX.X)	XX (XX.X)
Malaria1	XX (XX.X)	XX (XX.X)	XX (XX.X)
etc.	XX (XX.X)	XX (XX.X)	XX (XX.X)
**Physical examination**, n (%)			
Movement abnormality	XX (XX.X)	XX (XX.X)	XX (XX.X)
Skin abnormality	XX (XX.X)	XX (XX.X)	XX (XX.X)
Eyes abnormality	XX (XX.X)	XX (XX.X)	XX (XX.X)
Breathing abnormality	XX (XX.X)	XX (XX.X)	XX (XX.X)
Speech abnormality	XX (XX.X)	XX (XX.X)	XX (XX.X)
Hearing abnormality	XX (XX.X)	XX (XX.X)	XX (XX.X)
Other abnormality	XX (XX.X)	XX (XX.X)	XX (XX.X)

* at screening

4.3 ANALYSIS OF PRIMARY OUTCOME

Primary objective:
To compare the efficacy of the ACT artemether-lumefantrine versus a multivitamin preparation as defined by the 28-day PCR parasite positivity rate or incidence of confirmed clinical malaria of any species

Primary endpoints:
Composite endpoint
1. 28-day PCR Plasmodium positivity rate of any species or
2. Proportion of participants with confirmed clinical malaria of any species reported between day 0 and day 28

The main analysis strategy for the primary outcome will be the intention-to-treat (ITT) principle followed by the per protocol (PP) analysis. Thus, we will first analyse the ITT population in which all participants recruited in the trial will be included in the analysis according to the randomisation arm irrespective of what they actually got. These ITT analyses will be followed by the analysis of the per protocol (PP) population in which, participants who did not adhere to the protocol will be excluded from analysis.

The efficacy of the ACT artemether-lumefantrine versus a multivitamin preparation as defined by the 28-day PCR parasite positivity rate or incidence of confirmed clinical malaria analysis in each arm will be summarised using proportions and the 95% confidence intervals. Crude proportions will be calculated with the exact binomial 95% confidence intervals. The risks differences in efficacy between AL and multivitamin will be reported along with the corresponding 95% confidence intervals. The robust standard error will be used to adjust for intracluster correlation of episodes from the same individual. The mixed effects Poisson/negative binomial regression model will be used to model the adjusted incidence rate ratios (IRR). The mixed effects models will take into account the correlation of multiple episodes from same participant.

Tests of significance will be performed at 5% significance level.

4.3.1 Handling of missing data

For analyses of proportions, missing outcome will be imputed using plausible values. For example, the worst case scenario may be deemed appropriate but in that case sensitivity analysis would be done with the best case scenario.

4.3.2 Comparison of the efficacy of the ACT artemether-lumefantrine versus a multivitamin preparation as defined by the 28-day, 56-day, 84-day PCR parasite positivity rate or incidence of confirmed clinical malaria of any species

The efficacy of the artemether-lumefantrine versus a multivitamin as defined by the 28-day PCR parasite positivity rate or incidence of confirmed clinical malaria reported between day 0 and day 28 of any species from all follow-up months and for each follow-up month (day 28, day 56 and day 84) will be compared and presented in table 2 below.

Table 2 Comparison of efficacy of the ACT artemether-lumefantrine versus a multivitamin preparation as defined by the 28-day PCR parasite positivity or confirmed clinical malaria of any species

	Efficacy rate, n (%)		Risks difference/Incidence rate ratio (95% CI)	p-value
	AL (N=XXX)	Multivitamin (N=XXX)		
All months	XX (XX.X)	XX (XX.X)	XX.X (XX.X - XX.X)	X.XXX
Month 1	XX (XX.X)	XX (XX.X)	XX.X (XX.X - XX.X)	X.XXX
Month 2	XX (XX.X)	XX (XX.X)	XX.X (XX.X - XX.X)	X.XXX
Month 3	XX (XX.X)	XX (XX.X)	XX.X (XX.X - XX.X)	X.XXX

4.4 ANALYSIS OF SECONDARY OUTCOMES

4.4.1 Comparison of the efficacy of the ACT artemether-lumefantrine versus a multivitamin preparation As defined by the 28-day PCR parasite positivity or confirmed clinical malaria for each species

The efficacy of the artemether-lumefantrine versus a multivitamin as defined by the 28-day PCR parasite positivity rate and incidence of confirmed clinical malaria reported between day 0 and day 28 for each species from all follow-up months and for each follow-up month (day28, day 56 and day 84) will be compared and presented in table 3 below.

Table 3 Comparison of efficacy of the ACT artemether-lumefantrine versus a multivitamin preparation as defined by the 28-day PCR parasite positivity or confirmed clinical malaria for each species

	Efficacy rate, n (%)		Risks difference/Incidence rate ratio (95% CI)	p-value
	AL (N=XXX)	Multivitamin (N=XXX)		
P. faciparum				
All months	XX (XX.X)	XX (XX.X)	XX.X (XX.X - XX.X)	X.XXX
Month 1	XX (XX.X)	XX (XX.X)	XX.X (XX.X - XX.X)	X.XXX
Month 2	XX (XX.X)	XX (XX.X)	XX.X (XX.X - XX.X)	X.XXX
Month 3	XX (XX.X)	XX (XX.X)	XX.X (XX.X - XX.X)	X.XXX
P. vivax				
All months	XX (XX.X)	XX (XX.X)	XX.X (XX.X - XX.X)	X.XXX
Month 1	XX (XX.X)	XX (XX.X)	XX.X (XX.X - XX.X)	X.XXX
Month 2	XX (XX.X)	XX (XX.X)	XX.X (XX.X - XX.X)	X.XXX
Month 3	XX (XX.X)	XX (XX.X)	XX.X (XX.X - XX.X)	X.XXX
Other species *P. malariae, P. Ovale, P. knowlesi (PCR result only)*				
All months	XX (XX.X)	XX (XX.X)	XX.X (XX.X - XX.X)	X.XXX
Month 1	XX (XX.X)	XX (XX.X)	XX.X (XX.X - XX.X)	X.XXX
Month 2	XX (XX.X)	XX (XX.X)	XX.X (XX.X - XX.X)	X.XXX
Month 3	XX (XX.X)	XX (XX.X)	XX.X (XX.X - XX.X)	X.XXX

4.4.2 Quantifying the impact of the ACT artemether-lumefantrine (AL) as prophylaxis for forest goers on overall malaria transmission using mathematical modelling

A transmission dynamic mathematical model will be developed and used to simulate the impact of AL prophylaxis on malaria transmission at a population level.

4.4.3 Assessing the impact of artemether-lumefantrine prophylaxis on the spread of genetic markers of artemisinin (such as Kelch13 mutations) and partner drug resistance.

The prevalence of *Kelch13* mutations and other genetic markers of antimalarial drug resistance of known functional significance will be analysed and presented in table 4 below.

Table 4 Day 28 -PCR positivity by resistance markers e.g. *Pfkelch13* status

	Detection of relevant *Pfkelch13* mutation			
	Yes<br>(N=XXX)	No (wild type)<br>(N=XXX)	Risk Difference<br>(95% CI)	p-value
Artemether-Lumefantrine	XX.X (X.X)	XX.X (X.X)	XX.X (X.X)	X.XXX
Multivitamin	XX.X (X.X)	XX.X (X.X)	XX.X (X.X)	X.XXX

4.4.4 Details of place of residence, work, recent travel history and risk behaviours of forest goers in order to improve the understanding of high risk groups, locations of malaria transmission and possible routes spread of malaria and artemisinin resistance.

Data on the place of residence and work have been presented in table 1 above. The travel history and risk behaviours during the course of prophylaxis will be presented descriptively by study arm in table 5 below.

Table 5 Travel history and risk behaviours during the course of prophylaxis

	AL<br>(N=XXX)	Multivitamin<br>(N=XXX)	p-value
**Recent travel history**, n(%)			
**Travel destination**			
Stung Treng	XX (XX.X)	XX (XX.X)	0.XXX
Siem Pang	XX (XX.X)	XX (XX.X)	0.XXX
Others	XX (XX.X)	XX (XX.X)	0.XXX
**Travelling reasons**			
XX	XX (XX.X)	XX (XX.X)	0.XXX
XX	XX (XX.X)	XX (XX.X)	0.XXX
**Risk behaviours**			
Forest visit, n(%)	XX (XX.X)	XX (XX.X)	0.XXX
How many nights spent in the forest, median (IQR)	XX.X (XX.X - XX.X)	XX.X (XX.X - XX.X)	0.XXX
How many days spent in the forest, median (IQR)	XX.X (XX.X - XX.X)	XX.X (XX.X - XX.X)	0.XXX

Duration of travelling (days), median (IQR)	XX.X (XX.X - XX.X)	XX.X (XX.X - XX.X)	0.XXX
How many other people did you travel with? , median (IQR)	XX.X (XX.X - XX.X)	XX.X (XX.X - XX.X)	0.XXX
Sleep in the forest, n(%)	XX (XX.X)	XX (XX.X)	0.XXX
Bednet use, n(%)	XX (XX.X)	XX (XX.X)	0.XXX
Hammock net use, n(%)	XX (XX.X)	XX (XX.X)	0.XXX
Repellent spray or cream use, n(%)	XX (XX.X)	XX (XX.X)	0.XXX
Mosquito coil use, n(%)	XX (XX.X)	XX (XX.X)	0.XXX
Use anything else to protect you from mosquito bites, n(%)	XX (XX.X)	XX (XX.X)	0.XXX
Take any other antimalaria medicine, n(%)	XX (XX.X)	XX (XX.X)	0.XXX
Use anything else to protect you from getting malaria, n(%)	XX (XX.X)	XX (XX.X)	0.XXX

4.4.5 Adverse events and serious adverse events by study arms during the course of prophylaxis.

Adverse events will be graded according to Common Terminology Criteria for Adverse Events (CTCAE) Version 5.0 November 2017.

All adverse event summaries will refer to treatment emergent adverse events, i.e. adverse events that newly started or increased in intensity after the study drug administration. AE summaries will be generated for all AEs that occurred after study drug administration, until the end of the study.

Incidence of adverse events and serious adverse events by study arms during the course of prophylaxis will be presented in the table 6a below. AE intensity and relationship to study drug wil be presented as in tables 6b and 6c. Graphical presentations might be considered when presenting these.

Table 6a Adverse events

	Adverse event, n (%)		p-value
	AL<br>(N=XXX)	Multivitamin<br>(N=XXX)	
**Signs and symptoms**			
Headache	XX (XX.X)	XX (XX.X)	0.XXX
Loss of appetite	XX (XX.X)	XX (XX.X)	0.XXX
Dizziness	XX (XX.X)	XX (XX.X)	0.XXX
Fatigue	XX (XX.X)	XX (XX.X)	0.XXX
Joint pain	XX (XX.X)	XX (XX.X)	0.XXX
Muscle pain	XX (XX.X)	XX (XX.X)	0.XXX
Abdominal pain	XX (XX.X)	XX (XX.X)	0.XXX
Nausea	XX (XX.X)	XX (XX.X)	0.XXX
Vomiting	XX (XX.X)	XX (XX.X)	0.XXX
Diarrhoea	XX (XX.X)	XX (XX.X)	0.XXX
Itching	XX (XX.X)	XX (XX.X)	0.XXX

Cough	XX (XX.X)	XX (XX.X)	0.XXX
Fever	XX (XX.X)	XX (XX.X)	0.XXX
**Serious adverse events**	XX (XX.X)	XX (XX.X)	0.XXX

Table 6b Adverse events by severity

	AL					Multivitamin				
	Highest grade					Highest grade				
AE, n (%)	Mild	Moderate	Severe	Life-threatening	Fatal	Mild	Moderate	Severe	Life-threatening	Fatal
Headache	XX (XX.X)	XX (XX.X)	XX (XX.X)	XX (XX.X)	XX (XX.X)	XX (XX.X)	XX (XX.X)	XX (XX.X)	XX (XX.X)	XX (XX.X)
Loss of appetite	XX (XX.X)	XX (XX.X)	XX (XX.X)	XX (XX.X)	XX (XX.X)	XX (XX.X)	XX (XX.X)	XX (XX.X)	XX (XX.X)	XX (XX.X)
Dizziness	XX (XX.X)	XX (XX.X)	XX (XX.X)	XX (XX.X)	XX (XX.X)	XX (XX.X)	XX (XX.X)	XX (XX.X)	XX (XX.X)	XX (XX.X)
Fatigue	XX (XX.X)	XX (XX.X)	XX (XX.X)	XX (XX.X)	XX (XX.X)	XX (XX.X)	XX (XX.X)	XX (XX.X)	XX (XX.X)	XX (XX.X)
Joint pain	XX (XX.X)	XX (XX.X)	XX (XX.X)	XX (XX.X)	XX (XX.X)	XX (XX.X)	XX (XX.X)	XX (XX.X)	XX (XX.X)	XX (XX.X)
Muscle pain	XX (XX.X)	XX (XX.X)	XX (XX.X)	XX (XX.X)	XX (XX.X)	XX (XX.X)	XX (XX.X)	XX (XX.X)	XX (XX.X)	XX (XX.X)
Abdominal pain	XX (XX.X)	XX (XX.X)	XX (XX.X)	XX (XX.X)	XX (XX.X)	XX (XX.X)	XX (XX.X)	XX (XX.X)	XX (XX.X)	XX (XX.X)
Nausea	XX (XX.X)	XX (XX.X)	XX (XX.X)	XX (XX.X)	XX (XX.X)	XX (XX.X)	XX (XX.X)	XX (XX.X)	XX (XX.X)	XX (XX.X)
Vomiting	XX (XX.X)	XX (XX.X)	XX (XX.X)	XX (XX.X)	XX (XX.X)	XX (XX.X)	XX (XX.X)	XX (XX.X)	XX (XX.X)	XX (XX.X)
Diarrhoea	XX (XX.X)	XX (XX.X)	XX (XX.X)	XX (XX.X)	XX (XX.X)	XX (XX.X)	XX (XX.X)	XX (XX.X)	XX (XX.X)	XX (XX.X)
Itching	XX (XX.X)	XX (XX.X)	XX (XX.X)	XX (XX.X)	XX (XX.X)	XX (XX.X)	XX (XX.X)	XX (XX.X)	XX (XX.X)	XX (XX.X)
Cough	XX (XX.X)	XX (XX.X)	XX (XX.X)	XX (XX.X)	XX (XX.X)	XX (XX.X)	XX (XX.X)	XX (XX.X)	XX (XX.X)	XX (XX.X)
Fever	XX (XX.X)	XX (XX.X)	XX (XX.X)	XX (XX.X)	XX (XX.X)	XX (XX.X)	XX (XX.X)	XX (XX.X)	XX (XX.X)	XX (XX.X)

Table 6c Adverse events by relationship to study drug

	AL				Multivitamin			
	Relationship to drug				Relationship to drug			
AE, n (%)	Unrelated	Probably	Possibly	Definitely	Unrelated	Probably	Possibly	Definitely
Headache	XX (XX.X)	XX (XX.X)	XX (XX.X)	XX (XX.X)	XX (XX.X)	XX (XX.X)	XX (XX.X)	XX (XX.X)
Loss of appetite	XX (XX.X)	XX (XX.X)	XX (XX.X)	XX (XX.X)	XX (XX.X)	XX (XX.X)	XX (XX.X)	XX (XX.X)
Dizziness	XX (XX.X)	XX (XX.X)	XX (XX.X)	XX (XX.X)	XX (XX.X)	XX (XX.X)	XX (XX.X)	XX (XX.X)
Fatigue	XX (XX.X)	XX (XX.X)	XX (XX.X)	XX (XX.X)	XX (XX.X)	XX (XX.X)	XX (XX.X)	XX (XX.X)
Joint pain	XX (XX.X)	XX (XX.X)	XX (XX.X)	XX (XX.X)	XX (XX.X)	XX (XX.X)	XX (XX.X)	XX (XX.X)

Muscle pain	XX (XX.X)	XX (XX.X)	XX (XX.X)	XX (XX.X)	XX (XX.X)	XX (XX.X)	XX (XX.X)	XX (XX.X)
Abdominal pain	XX (XX.X)	XX (XX.X)	XX (XX.X)	XX (XX.X)	XX (XX.X)	XX (XX.X)	XX (XX.X)	XX (XX.X)
Nausea	XX (XX.X)	XX (XX.X)	XX (XX.X)	XX (XX.X)	XX (XX.X)	XX (XX.X)	XX (XX.X)	XX (XX.X)
Vomiting	XX (XX.X)	XX (XX.X)	XX (XX.X)	XX (XX.X)	XX (XX.X)	XX (XX.X)	XX (XX.X)	XX (XX.X)
Diarrhoea	XX (XX.X)	XX (XX.X)	XX (XX.X)	XX (XX.X)	XX (XX.X)	XX (XX.X)	XX (XX.X)	XX (XX.X)
Itching	XX (XX.X)	XX (XX.X)	XX (XX.X)	XX (XX.X)	XX (XX.X)	XX (XX.X)	XX (XX.X)	XX (XX.X)
Cough	XX (XX.X)	XX (XX.X)	XX (XX.X)	XX (XX.X)	XX (XX.X)	XX (XX.X)	XX (XX.X)	XX (XX.X)
Fever	XX (XX.X)	XX (XX.X)	XX (XX.X)	XX (XX.X)	XX (XX.X)	XX (XX.X)	XX (XX.X)	XX (XX.X)

4.4.6 Prevalence of asymptomatic Plasmodium infections in high risk populations at varying seasonal time points

Prevalence of asymptomatic Plasmodium at baseline, stratified by season and risk factors will be presented in table 7 below. The numbers of asymptomatic Plasmodium infections will be visuallized by follow-up months and study arm in figure 2 below.

Table 7 Prevalence of asymptomatic Plasmodium infection at baseline

	Prevalence of asymptomatic Plasmodium infection at baseline, n (%)		
	AL (N=XXX)	Multivitamin (N=XXX)	Total
Overall	XX (XX.X)	XX (XX.X)	XX (XX.X)
Season			
Month 1	XX (XX.X)	XX (XX.X)	XX (XX.X)
Month 2	XX (XX.X)	XX (XX.X)	XX (XX.X)
Month 3	XX (XX.X)	XX (XX.X)	XX (XX.X)
Riskfactor1			
Category1	XX (XX.X)	XX (XX.X)	XX (XX.X)
Category2	XX (XX.X)	XX (XX.X)	XX (XX.X)
Riskfactor2			
Category1	XX (XX.X)	XX (XX.X)	XX (XX.X)
Category2	XX (XX.X)	XX (XX.X)	XX (XX.X)
Riskfactor3			
Category1	XX (XX.X)	XX (XX.X)	XX (XX.X)
Category2	XX (XX.X)	XX (XX.X)	XX (XX.X)

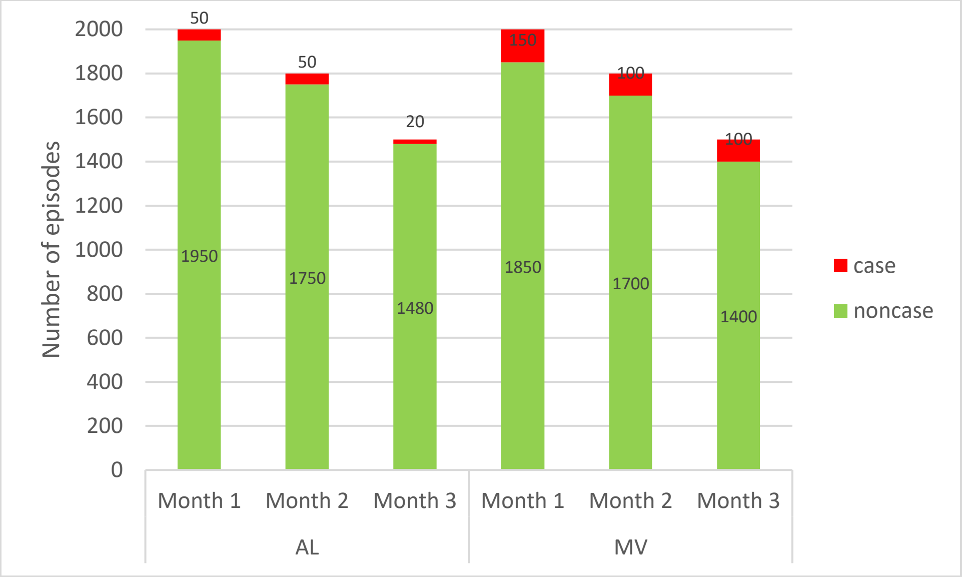

Figure 2 Number of asymptomatic Plasmodium cases by follow-up months

AMENDMENT TO THE ORGINAL ANALYSIS PLAN

We initially planned that missing data will be imputed using plausible values in our original SAP (and best/worst case scenarios in the protocol). However, at the time of analysis we noted that there is an increasing body of literature that suggests that Multiple Imputation (MI) should not be considered as the only way of addressing missing data (Sullivan et al 2018). Recent literature further indicates that more often, the Complete Case analysis performs comparably to MI in randomized controlled trials and sometimes even better than MI (Groenwold et al 2012; Mukaka et al 2016; Sullivan et al 2018). Suggested reasons for the good performance of complete case analysis are that data are possibly missing completely at random (MCAR) and in that case, MI may introduce additional variability that tends to bias estimates. Because of this increasing support in literature for the use of complete case analysis, we thought of using complete case analysis for ITT as this approach was being recommended by experts in methods of missing data whom we contacted prior to analysis. Our assumption is that the outcome data in our study is likely to be missing completely at random. When data is MCAR, complete case analysis performs better that extreme case (best/worst case) analysis.

Reference

Groenwold, R. H., Donders, A. R. T., Roes, K. C., Harrell Jr, F. E., & Moons, K. G. (2012). Dealing with missing outcome data in randomized trials and observational studies.*American journal of epidemiology*,*175*(3), 210-217.

Mukaka, M., White, S. A., Terlouw, D. J., Mwapasa, V., Kalilani-Phiri, L., & Faragher, E. B. (2016). Is using multiple imputation better than complete case analysis for estimating a prevalence (risk) difference in randomized controlled trials when binary outcome observations are missing?.*Trials*,*17*(1), 1-12.

Sullivan, T. R., White, I. R., Salter, A. B., Ryan, P., & Lee, K. J. (2018). Should multiple imputation be the method of choice for handling missing data in randomized trials?.*Statistical methods in medical research*,*27*(9), 2610-2626.

Pimnara Peerawaranun 13th June 2022

Print name & signature, Statistician Date

Prof Mavuto Mukaka 13th June 2022

Print Name & signature, Head of Statistics Date

Prof Lorenz von Seidlein 16th June 2022

Print Name & signature, Investigator Date

Prof Richard Maude 18th June 2022

Print Name & signature, Principal Investigator Date
